# Could widespread use of antiviral treatment curb the COVID-19 pandemic? A modeling study

**DOI:** 10.1186/s12879-022-07639-1

**Published:** 2022-08-09

**Authors:** Laura Matrajt, Elizabeth R. Brown, Myron S. Cohen, Dobromir Dimitrov, Holly Janes

**Affiliations:** 1 Vaccine and Infectious Disease Division, Fred Hutchinson Cancer Center, Seattle, USA; 2 Department of Biostatistics, University of Washington, Seattle, USA; 3 Institute of Global Health and Infectious Diseases, University of North Carolina at Chapel Hill, Chapel Hill, USA; 4 Department of Applied Mathematics, University of Washington, Seattle, USA

**Keywords:** Antiviral treatment, COVID-19, SARS-CoV-2, Mathematical model, Agent-based model

## Abstract

**Background:**

Despite the development of safe and effective vaccines, effective treatments for COVID-19 disease are still urgently needed. Several antiviral drugs have shown to be effective in reducing progression of COVID-19 disease.

**Methods:**

In the present work, we use an agent-based mathematical model to assess the potential population impact of the use of antiviral treatments in four countries with different demographic structure and current levels of vaccination coverage: Kenya, Mexico, United States (US) and Belgium. We analyzed antiviral effects on reducing hospitalization and death, and potential antiviral effects on reducing transmission. For each country, we varied daily treatment initiation rate (DTIR) and antiviral effect in reducing transmission (AVT).

**Results:**

Irrespective of location and AVT, widespread antiviral treatment of symptomatic adult infections (20% DTIR) prevented the majority of COVID-19 deaths, and recruiting 6% of all adult symptomatic infections daily reduced mortality by over 20% in all countries. Furthermore, our model projected that targeting antiviral treatment to the oldest age group (65 years old and older, DTIR of 20%) can prevent over 30% of deaths. Our results suggest that early antiviral treatment (as soon as possible after inception of infection) is needed to mitigate transmission, preventing 50% more infections compared to late treatment (started 3 to 5 days after symptoms onset). Our results highlight the synergistic effect of vaccination and antiviral treatment: as the vaccination rate increases, antivirals have a larger relative impact on population transmission. Finally, our model projects that even in highly vaccinated populations, adding antiviral treatment can be extremely helpful to mitigate COVID-19 deaths.

**Conclusions:**

These results suggest that antiviral treatments can become a strategic tool that, in combination with vaccination, can significantly reduce COVID-19 hospitalizations and deaths and can help control SARS-CoV-2 transmission.

**Supplementary Information:**

The online version contains supplementary material available at 10.1186/s12879-022-07639-1.

## Background

With over 5 million deaths worldwide [1], the COVID-19 pandemic has proven difficult to contain. Despite the development, advent, licensure, and rollout of many safe and effective vaccines [2], controlling SARS-CoV-2 transmission has shown to be elusive for several reasons, including vaccine supply shortages in low- and middle-income countries [3], vaccine hesitancy [4], and the emergence of new variants [5]. Indeed, the Delta and Omicron variants, that emerged in summer and fall of 2021, quickly became the predominant strains and have caused large epidemic outbreaks, even in highly vaccinated regions [1]. Rapidly producing such COVID-19 vaccines has been an amazing scientific endeavor, but effective tools to treat COVID-19 disease are still urgently needed. Monoclonal antibodies, antibody cocktails and antiretroviral treatments have been, and continue to be studied to treat SARS-CoV-2 infection and to prevent progression to severe disease [6]. Several treatments have been found to reduce hospitalizations by 30 to 89% [7–10] when taken within the first five days after developing symptoms. Some of them are approved for early treatment of patients with mild-to moderate COVID-19 who are at high-risk of progression to severe disease while others are approved for hospitalized patients, with one approved as a pre-exposure prophylaxis [11]. Furthermore, most studies have shown that these antiviral treatments significantly reduced the amount of infectious virus in the nasal mucosa of treated individuals [12, 13]. Hence, the advent of effective antiviral drugs raises the possibility that in treating infected individuals we may reduce onward transmission (indirect population benefit) while also protecting the treated person from severe disease (direct benefit). The use of antiviral treatments as an effective means of prevention and epidemic control is not new. During the 2009 influenza A H1N1 pandemic, just a few weeks after the first case of influenza A H1N1 was identified in the US, the US government released 11 million courses of antiviral drugs for influenza (25% of the antiviral supply) from the National Stockpile as a potential tool to control transmission and mitigate disease [14]. Treatment as Prevention is considered a primary method of epidemic control for HIV, as research has demonstrated that earliest detection and treatment suppressing HIV replication stops secondary transmission while having the the greatest effect at the individual level [15–17].

Over the past several months, the availability of antigen tests has expanded considerably, facilitating the early diagnosis of SARS-CoV-2 infection and possible early treatment [18, 19]. The US government has purchased 20 million courses of the antiviral pill paxlovid; these are expected to be delivered in early 2022. Furthermore, a “Test to Treat” initiative was recently announced as part of a new phase in the US government pandemic response [20]. The Medicines Patent Pool and the manufacturers of molnupiravir and paxlovid (Merck and Pfizer respectively) have announced license agreements to facilitate global access for these drugs [21, 22], and Pfizer will donate 4 million courses of paxlovid to UNICEF for use in lower-income countries in the following months [23]. Hence, it is possible that in the next few months antiviral treatments will become widely available globally.

In this work, we use an agent-based mathematical model to evaluate the potential population impact of widespread use of antiviral treatments in reducing hospitalization risk and population-level transmission. We explored the use of antiviral treatments in four different countries (Kenya, Mexico, US and Belgium) with very different demographic composition and vastly different proportions of vaccinated individuals.

We showed that the synergistic use of vaccine and antiviral treatments can significantly reduce the burden of COVID-19. Further, our model suggested that targeted use of antiviral treatments can be used to prevent the majority of deaths.

## Results

Briefly, we used COVASIM, a previously developed agent-based model of SARS-CoV-2 spread calibrated to Seattle, WA [24, 25]. Our model simulates a population of 500,000 people interacting through a network over the course of 6 months, where each individual in the population is an agent. Every day, individuals contact others in four possible locations: home, school, work or community. At a given point in time, individuals can be susceptible, infected asymptomatic, pre-symptomatic or symptomatic, and recovered. Symptomatic infected individuals can develop a mild, severe or critical infection after symptoms onset. A fixed age-dependent proportion of infections is assumed to remain asymptomatic. Within each age stratum, asymptomatic infections are assumed 30% less transmissible than symptomatic infections (Additional file 1: Table S1). Infectivity is time dependent, being highest around symptom onset and decreasing afterwards. We assumed that 40% of the population has been infected in previous epidemic waves and protected from re-infection over the study duration, and compared these results to scenarios assuming 20 and 60% pre-existing immunity. We simulated populations of equal size in four different countries: Kenya, Mexico, US and Belgium. For each country, the model uses country-specific demographics to inform population structure and household sizes. Because we are interested in investigating population effects of antivirals in different epidemic contexts that may modulate their effects, we explored two main scenarios: first, we assumed deployment would take place under an Omicron-like wave, representing a high transmissible variant for which vaccines might not be very effective at preventing infections. In this scenario, we assumed vaccination coverages in each country as of January 3rd 2022, separating the proportion of the population that received full doses or boosters in US and Belgium. Then, we repeated the analysis assuming antiviral deployment under a Delta-like wave, a less transmissible variant for which vaccines remained moderately effective in preventing infections. Here, we assumed vaccination coverage as of October 12th, 2021. For each scenario, we used different parameters for viral transmission, and vaccine effectiveness (Methods for full details, Additional file 1: Table S2). Additional file 1: Figs. S1 and S2 highlight the effect of the population structure in the epidemic dynamics: Kenya, the country with the youngest population, has a lower number of cumulative deaths than Belgium, the country with the oldest population, despite having a much higher number of cumulative infections and a much lower vaccination coverage.

We assumed that the antiviral treatment would have two primary effects. First, in line with results from the EPIC-HR (paxlovid), PINETREE (remdesivir), COMET-ICE (sotrovimab), and MOVe-OUT (molnupiravir) clinical trials, that showed a very high reduction in hospitalization or deaths, we assumed an antiviral effect on hospitalization (denoted by $\text {AVH}$) by which a course of antiviral treatment would reduce the rate of hospitalization of symptomatic infected individuals by 88% (main results) or 30% (sensitivity analysis) [7–10]. Second, because clinical trials have reported an antiviral effect on viral load, [7, 12, 13], we assumed that the antiviral treatment would have an effect in overall transmission (denoted by $\text {AVT}$). Given uncertainty in how a reduction in viral load translates into a reduction in transmission, we explored reductions in secondary transmission 25, 50, 75, or 100% due to antiviral treatment.

We assumed that antiviral treatment would be given to symptomatically infected adults (18 years or older) within 5 days from symptoms onset. We explored scenarios with treatment initiated within 5 days, within two days or between days 3 and 5 from symptoms onset, to study the impact of treatment timing on the results. To reflect real-world constraints on resources and infrastructure, we also varied the rate at which symptomatic adults could be identified and recruited for treatment, considering scenarios with a daily treatment initiation rate (DTIR) of 2, 4, 6, 8, 10, 20, $\dots$ 100% of symptomatic adults. For example, a 6% DTIR resulted on treating roughly 26% of the overall adult symptomatic infections (total symptomatic infections over the duration of the study), while a DTIR of 40% resulted in treating over 90% of the overall adult symptomatic infections. To capture variability, 100 simulations were run for each scenario. Throughout the text, we present the median percentage deaths and infections averted over the ensemble simulations and compared them to base-case scenario assuming that existing non-pharmaceutical measures remain in place for the next 6 months without additional vaccination. Cumulative deaths and infections (and respective confidence bounds) are presented in the Additional file 1.

**Fig. 1:**
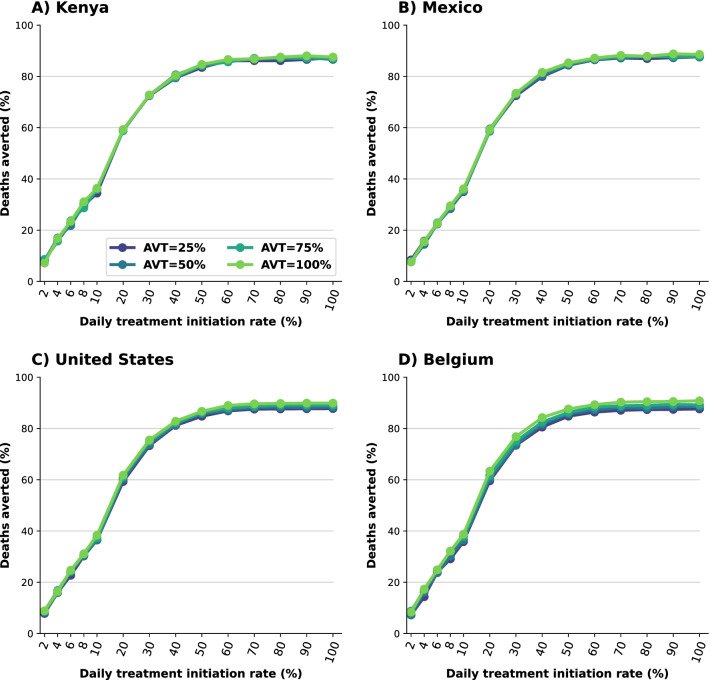
Percentage of deaths averted for **A** Kenya, **B** Mexico, **C** United States and **D** Belgium. Percentage of deaths averted assuming an epidemic wave with parameters similar to those of the Omicron epidemic wave (transmissibility, vaccine effectiveness, and vaccination coverage) when compared to a baseline of no antiviral treatment. For each country, the colors represent four possible values of AVT (25, 50, 75 or 100% reduction in viral transmission in treated symptomatic individuals) and a daily treatment initiation rate (DTIR) of 2–100% of adult symptomatic individuals within the first 5 days of symptoms

**Fig. 2:**
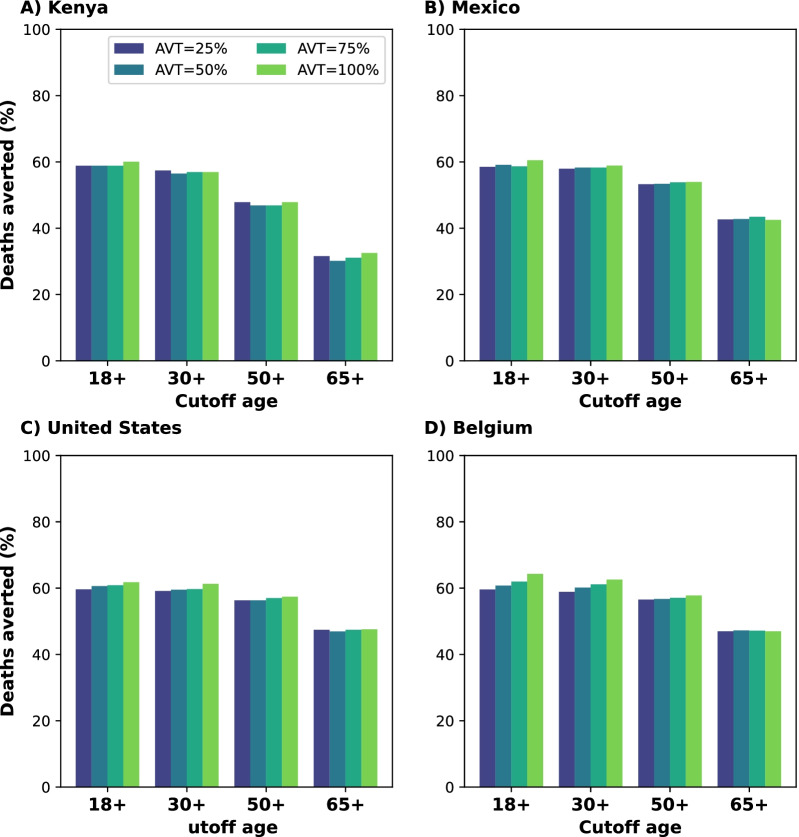
Percentage of deaths averted for targeted interventions for **A** Kenya, **B** Mexico, **C** United States and **D** Belgium. Percentage of deaths averted assuming an epidemic wave with parameters similar to those of the Omicron epidemic wave (transmissibility, vaccine effectiveness, and vaccination coverage) when compared to a baseline of no antiviral treatment using antiviral treatment in targeted age groups. For each country, the colors represent four possible values of AVT (25, 50, 75 or 100% reduction in viral transmission in treated symptomatic individuals) and targeting the antiviral treatment to symptomatic adults older than 18, 30, 50 or 65 years of age with a daily treatment initiation rate of 20%

### Omicron-like wave

*Targeted use of antiviral treatment can avert large numbers of deaths.* We first examine the use of antiviral treatment in all adults. For all the countries considered, irrespective of the antiviral effect on viral transmission, large numbers of deaths were averted for all scenarios, even if the DTIR was low. A 6% DTIR among all adult symptomatic infections averted over 20% of deaths when compared to no antiviral use in all countries (23, 23, 23 and 24% deaths averted for Kenya, Mexico, US and Belgium respectively assuming AVT = 25%). Over 58% of deaths would be averted if 20% of all adult symptomatic infections were identified and recruited for treatment daily (58, 58, 59 and 59% of deaths averted for Kenya, Mexico, US and Belgium respectively assuming AVT = 25%). If the DTIR increased to 50% or more, over 83% of deaths would be averted in all countries. We observed minimal differences between countries and assumed levels of antiviral reduction in transmission, pointing to the fact that these reductions are a result of direct rather than indirect antiviral treatment protection (Fig. 1 and Additional file 1: Fig. S1).

Because identifying, recruiting and treating large numbers of all the eligible symptomatic adults (individuals aged 18 or older) might be difficult to implement in practice, especially in countries where testing is not widely available or where antiviral treatment is in short supply, we then evaluated additional targeted strategies where we restricted antiviral treatment to adults over 30, 50 or 65 years of age and considered a 20% DTIR among these populations. Concentrating antiviral treatment in the older age groups can achieve high population impact: despite the fact that the older age groups are the smallest and have the highest vaccination rates (Additional file 1: Table S4), identifying and treating 20% of the daily symptomatic infections among older adults (aged 65 years old and older) averted 32, 43, 47 and 47% of all deaths in Kenya, Mexico, US and Belgium respectively (assuming 25% reduction in viral transmission). Adults aged 50 and older represent 26, 40 and 44% of the adult populations in Mexico, US and Belgium respectively, but recruiting 20% of the daily symptomatic infections in this age group averted the majority of deaths in these countries (53, 56 and 56% deaths averted in Mexico, US and Belgium respectively). More impressively, while this age group represents only 13% of the adult population in Kenya, initiating antiviral treatment in 20% of the symptomatic infections in this age group daily resulted in 47% of deaths averted. Extending the use of antiviral treatment to younger adults resulted in a marginal gain in Mexico, US and Belgium (a maximum 7, 4 and 6% more deaths averted respectively) when compared with targeted treatment in adults older than 50 (Fig. 2).

**Fig. 3:**
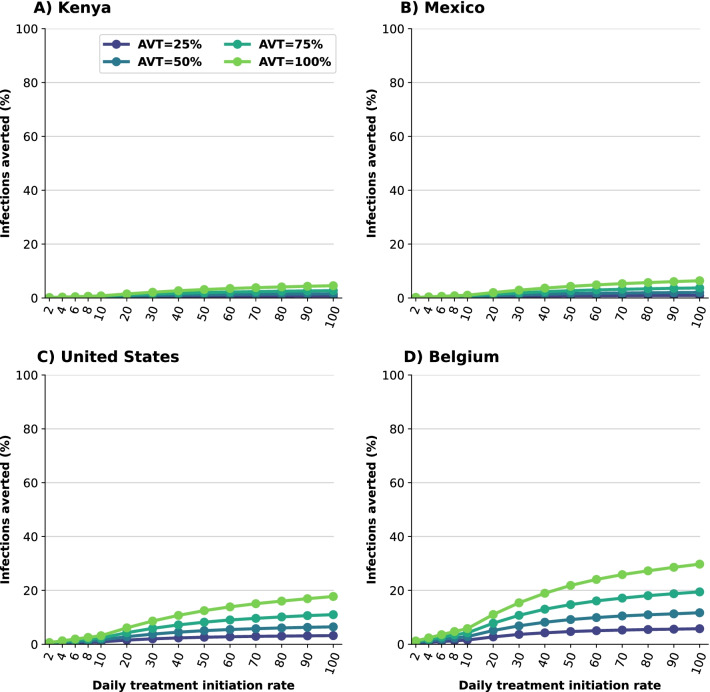
Percentage of infections averted for **A** Kenya, **B** Mexico, **C** United States and **D** Belgium. Percentage of infections averted (compared to a baseline of no antiviral treatment) assuming an epidemic wave with parameters similar to those of the Omicron epidemic wave (transmissibility, vaccine effectiveness, and vaccination coverage). For each country, the colors represent four possible values of AVT (25, 50, 75 or 100% reduction in viral transmission in treated symptomatic individuals) and a daily treatment initiation rate (DTIR) of 2–100% of adult symptomatic individuals within the first 5 days of symptoms

**Fig. 4:**
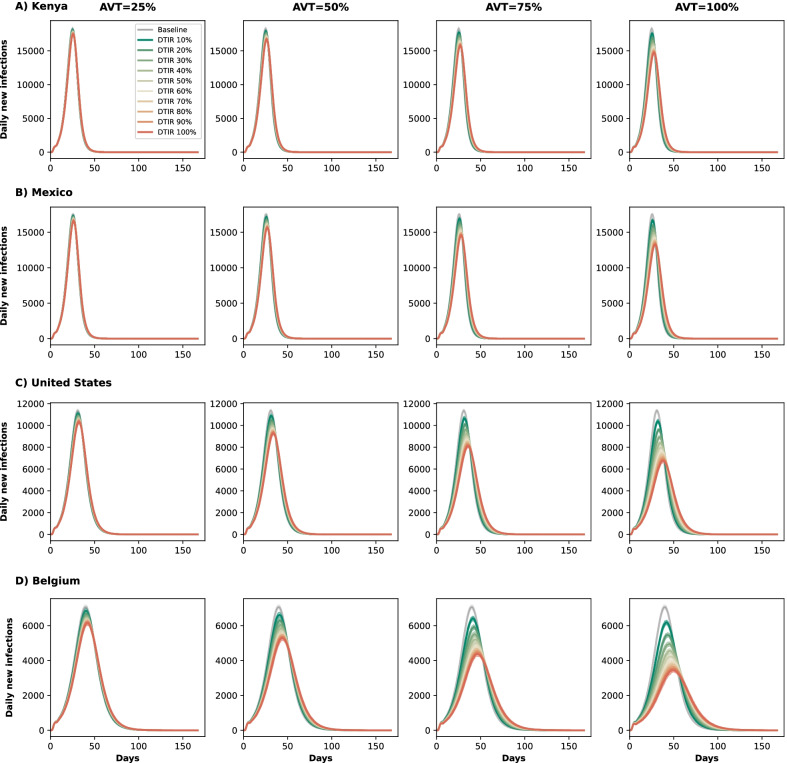
Epidemic curves assuming antiviral treatment coverage of 10–100% of eligible symptomatic individuals. Daily new infections assuming no antiviral treatment (Baseline) or assuming coverage of 10–100% of eligible symptomatic individuals in **A** Kenya, **B** Mexico, **C** United States and **D** Belgium. Here, we assumed an epidemic wave with parameters similar to those of the Omicron epidemic wave (transmissibility, vaccine effectiveness, and vaccination coverage). For each location, each column represents a different value of AVT (25, 50, 75 or 100% reduction in viral load)

*Synergistic effect of antiviral treatment and vaccine can mitigate SARS-CoV-2 transmission.* We evaluated the potential effect of antiviral treatment on population incidence of SARS-CoV-2, mediated through reduced transmission. Our model projected that antiviral treatment will have a very limited impact in mitigating transmission in the presence of a variant as transmissible as Omicron in countries like Kenya or Mexico that have low vaccination and boosting rates. In highly vaccinated countries like the US and Belgium, our results showed that antiviral treatment had some impact provided that a large proportion of symptomatic infections are identified and recruited for treatment and the antiviral effect on transmission is high (Fig. 3 and Additional file 1: Fig. S2). This is because the Omicron variant was very transmissible and vaccination with two doses had a minimal effect against preventing infection, but adding a third dose of vaccine provided some protection against acquisition. Further, we found a clear synergy of combining antiviral treatment and vaccination. While Belgium and the US have similar population structure, vaccination coverage in Belgium has been much higher than in the US (both with full doses and with boosters, Additional file 1: Tables S5 and S6). When combined with antiviral treatment, the difference in the vaccinated population had a big effect in mitigating transmission: for example, in the most optimistic scenario, assuming that the antiviral treatment completely blocked transmission, a 30% DTIR of adult symptomatic infections averted 8% of overall infections in the US, and almost twice as many infections were averted in Belgium (15%). In contrast, for this scenario, only 3% of overall infections were averted in Mexico, which had a high vaccination coverage but had not been boosted the population at that time, and 2% of the overall infections were averted in Kenya. Indeed with very limited vaccine supply, the effective reproductive number in Kenya is much larger than in the other countries, resulting in epidemics that are more difficult to control (Figs. 3 and 4). As expected, the antiviral effect on viral transmission played a bigger role in controlling SARS-CoV-2 transmission. With a low AVT (25%), a very limited number of infections were averted, irrespective of treatment initiation rate or country, with a maximum of 6% of infections averted, but a maximum of 30% of infections were averted with high AVT (Belgium, Fig. 3D). Note that, for AVT= 25, 50 or 75%, the number of infections averted plateaued for medium and high antiviral treatment coverage. For example, in the US, a DTIR of 50% of adult symptomatic infections with an antiviral treatment with AVT=50% averted 5% of infections, and treating an additional 20% of symptomatic adults per day (70% DTIR) resulted in only 1% more infections averted (Fig. 3).

**Fig. 5:**
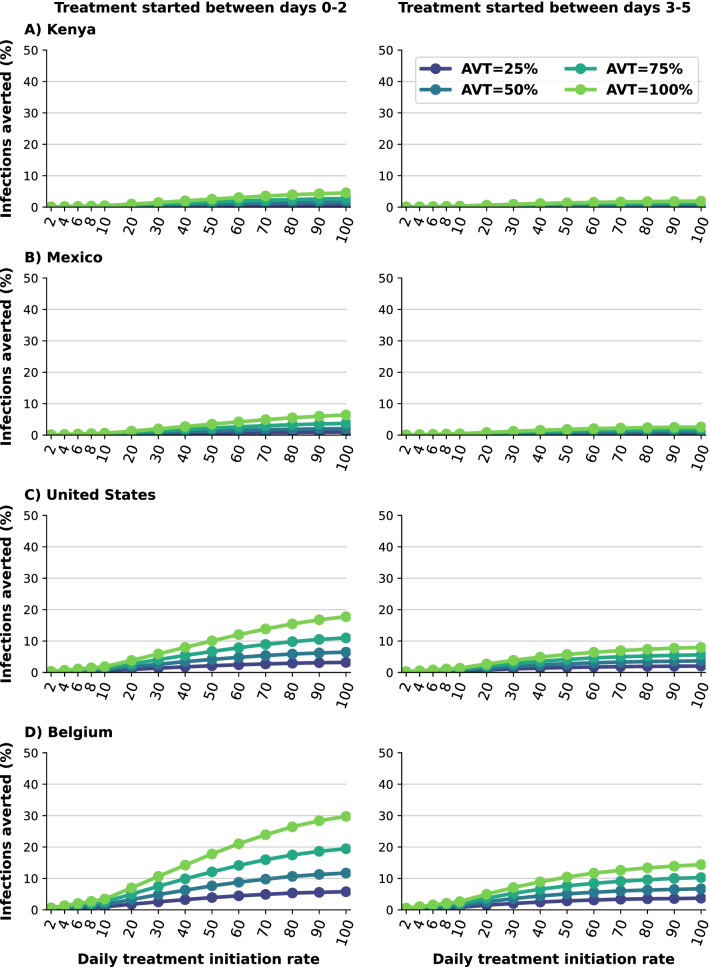
Percentage of deaths averted assuming early or late treatment initiation. Percentage of deaths averted (compared to a baseline of no antiviral treatment) for **A** Kenya, **B** Mexico, **C** United States and **D** Belgium for early (within the first two days of symptoms, left column) or late (between days 2 and 5 of symptoms, right column) treatment. Here, we assumed an epidemic wave with parameters similar to those of the Omicron epidemic wave (transmissibility, vaccine effectiveness, and vaccination coverage). For each country, the colors represent four possible values of AVT (25, 50, 75 or 100% reduction in viral transmission in treated symptomatic individuals) and covering 10–100% of eligible symptomatic individuals

*Early treatment is needed to mitigate transmission.* We analyzed the difference between early and late treatment by considering treating eligible symptomatic infections within the first two days of symptoms or alternatively between days 3 and 5 after symptom onset. For Kenya and Mexico, late treatment had no impact on transmission (Fig. 5A, B). This is because viral transmission is extremely high and antiviral treatment is happening after most of the secondary infections among symptomatic individuals have occurred. For the US or Belgium, starting antiviral treatment during the first two days of symptom onset prevented roughly twice as many subsequent infections compared to late treatment (Fig. 5C, D). For example, with a DTIR of 50% of symptomatic infections, and AVT = 75%, 12% and 7% of overall infections were averted in Belgium with early and late treatment respectively (Fig. 5D).

### Delta-like epidemic wave

In this section we present the results assuming antiviral treatment was given under similar conditions as those experienced during the Delta wave: we considered vaccination coverages as of October 12th, 2021, higher vaccine efficacy (Additional file 1: Table S2), and a virus as transmissible as the Delta variant (full details in Methods). Under this scenario, the number of deaths averted was slightly higher than for the Omicron wave with an average 2% more deaths averted in all countries (Fig. 1 and Additional file 1: Fig. S3). Additionally, there were some differences in the number of deaths averted depending on the assumed antiviral effect on transmission, with a maximum of 3, 4, 7 and 12% more deaths averted in Kenya, Mexico, US and Belgium respectively for an antiviral treatment fully blocking transmission compared with one reducing transmission by 25%, suggesting a small indirect effect on the number of deaths averted as a result of reduced transmission. More importantly, under this scenario, we observed that antiviral treatment markedly cut overall viral transmission. This is because the reduced transmissibility of a Delta-like variant combined with higher vaccine efficacy and higher proportions of the population vaccinated result in a lower effective reproductive number. For example, assuming a DTIR of 30% of symptomatic adults and AVT = 50%, 3, 7, 13 and 22% of infections were averted in Kenya, Mexico, US and Belgium respectively (compared to 1, 1, 4 and 7% under an Omicron wave respectively), Figs. 3 and Additional file 1: Fig. S4. Finally, the synergy between vaccination and antiviral treatment was more apparent. For example, Kenya and Mexico have a young population structure but Mexico had a much higher vaccination rate under this scenario, resulting in twice as many infections averted in Mexico compared to Kenya (7% vs 3% respectively, assuming DTIR = 30% and AVT = 50%). Similarly, the US and Belgium have an older population, with 40 and 44% of adults over 50 years old, but a higher proportion of the population was vaccinated in Belgium, resulting in roughly twice as many infections averted (13% vs 22%, respectively, assuming DTIR=30% and AVT=50%), Additional file 1: Fig. S4.

### Sensitivity analysis

In this section we analyze the sensitivity of our results to key model parameters. All the results presented here assumed an Omicron-type epidemic. Increase or decreased infection-acquired pre-existing immunity. Different places, even within a country, have experienced the COVID-19 pandemic in different ways, depending on local non-pharmaceutical interventions, culture (e.g. vaccine hesitancy, adherence to mask use) and population demographics [26, 27]. Hence, we considered the effect of pre-existing infection-acquired immunity on our results, assuming a lower (20%) or higher (60%) fraction of the population previously infected and currently immune. We observed minimal differences in the number of deaths averted for lower or higher assumed infection-acquired pre-existing immunity. This is expected, as the majority of deaths averted in our model are the result of direct effect of the antiviral treatment. However, the model projected differences in transmission assuming lower or higher infection-acquired pre-existing immunity. Lower infection-acquired immunity resulted in bigger epidemic waves and lower proportions of infections averted with antiviral treatment compared to the main results for all countries, all coverages and all AVEs (maximum 2, 3, 10 and 17% infections averted in Kenya, Mexico, US and Belgium respectively), Additional file 1: Figs. S5 (left column) and S6. In contrast, higher infection-acquired immunity resulted in lower epidemic curves and more infections averted (maximum 12, 17, 39 and 63% infections averted in Kenya, Mexico, US and Belgium respectively), Additional file 1: Figs. S5 (right column) and S7.

*Reduced antiviral effectiveness against hospitalization,* $\text {AVH}$ = 30%. One antiviral treatment (molnupiravir) was shown to have a 30% reduction in hospitalizations and deaths [7], so we repeated our analysis assuming this lower value for $\text {AVH}$. As expected, an antiviral treatment which reduces the hospitalization risk of symptomatically infected by 30% averted considerably less deaths than our main scenario (with $\text {AVH}$ = 88%). For example, a DTIR of 20% among adult symptomatic infections resulted in over 20% of the deaths averted for all four countries, a $\sim$40% reduction compared to the main scenario. Under this scenario, there was a stronger synergistic effect of vaccination and with the antiviral effect both on deaths averted and on transmission that was compounded with the antiviral effect on transmission: for example, for a DTIR of 20% and AVT=25% there were 20% and 22% deaths averted in Kenya and Belgium respectively (2% more deaths averted in Belgium), but if AVT=100% there was a 9% difference between the number of deaths averted in Belgium and Kenya (22% and 31% deaths averted in Kenya and Belgium respectively) difference, Additional file 1: Fig. S8.

*Decrease in asymptomatic infectiousness* The relative infectivity of asymptomatic individuals remains uncertain [28]. We explored scenarios in which asymptomatic individuals are 50% less infectious than symptomatic individuals. The overall antiviral effect on mortality was not sensitive to this parameter: our model projected nearly identical deaths averted for each scenario considered, Additional file 1: Fig. S9. However, the number of infections averted assuming a lower infectivity of asymptomatic infections was higher, with higher differences observed in countries with higher vaccination coverages. For example, under this scenario, 20% of the infections were averted in the US assuming a DTIR of 50% and an antiviral treatment fully reducing transmission, compared with 12% infections averted in the main scenario, Additional file 1: Fig. S10.

## Discussion

Despite the existence of effective vaccines, the number of deaths due to COVID-19 globally is still above 7000 per day, with over 1000 deaths per day in the US [1], due to issues with supply, hesitancy and logistics. This highlights the need of effective and affordable treatment options. Several antiviral treatments have shown to be highly efficacious against COVID-19 hospitalization and death, provided that treatment is started early, within 5 days of symptoms onset [7–10]. In the present work we analyzed the potential population impact of antiviral treatments for reducing SARS-CoV-2 transmission and COVID-19 related hospitalizations and deaths. We considered four equal-sized populations from four different countries (Kenya, Mexico, US and Belgium), representing different population structures and vaccination coverage. We further explored the impact of this intervention under epidemic waves with parameters similar to the Delta and Omicron waves. Our results suggest that irrespective of country, AVT and variant, widespread use of antiviral treatment (daily treatment initiation rate of 20% of the daily adult symptomatic infections) could prevent the majority of deaths. We projected that under an Omicron-like epidemic wave, with extremely high viral transmissibility and low vaccine effectiveness against infection acquisition, antiviral treatment of symptomatic infections will have very limited impact on transmission. This is expected as a large proportion of the infection chain is occurring in asymptomatic infections, and treating symptomatic infections under high transmissibility is not sufficient to curb overall transmission. Under this scenario, expanding antiviral treatment access to all detected infections might increase the effectiveness of antiviral treatments in this regard. However, if newer variants behaved more like the Delta variant, then our model showed that antiviral treatment in symptomatic adults can have a larger impact on curbing overall infections. Considerable practical challenges would be faced in identifying, testing, and treating symptomatic infections among all adults, especially among the younger adults, who are at much lower risk to progress to severe disease and death. Indeed, test availability and acceptance, timely report of test results and identification of infected individuals, have all proven to be difficult obstacles to overcome at different times during the previous months.

A clinical outcome of considerable interest is long COVID, to which all age groups appear to be susceptible [29, 30]. Long COVID consists of persistent symptoms including abnormal breathing, headache, fatigue, muscle weakness, anxiety or depression, headache, myalgia, and cognitive dysfunction [31]. To date, it is not known if, how and which (if any) antiviral treatments are effective against any of these post-COVID conditions. If antiviral treatments (either monoclonal antibodies or antiviral chemotherapy) were shown to be effective against long COVID, this could justify widespread use of antiviral treatments among all strata of the population. Of course, continuing promoting and financing wide scale testing would be critical for achieving widespread antiviral use. Moreover, our model suggests that there is a synergistic effect of combining antiviral treatment and vaccination: countries with larger proportion of their populations vaccinated are expected to benefit more from adding antiviral treatment to their pandemic control toolbox. This emphasizes the need of continuous effort to improve vaccination coverage especially in settings where it is extremely low. Finally, our simulations showed that early treatment, when viral transmission is highest [32, 33], is important for mitigating transmission and could be used as a prevention tool. This is in agreement with previous work [34–38] that has shown the potential use of antiviral therapies to reduce COVID-19 related mortality.

Our model, like all mathematical models, is subject to several limitations. We did not consider the development of antiviral resistance, yet it is possible that if antiviral treatments are widely used, resistance could rapidly develop. In fact, monoclonal antibody treatments that were highly effective against older variants have become ineffective against newer ones [39, 40]. However, small molecule oral antiviral treatments have been shown to remain efficacious against new variants [41]. We analyzed the use of antiviral treatment in symptomatic individuals, and did not explore its use in asymptomatic infections or as a prophylaxis. Further, we assumed that an antiviral effect reducing transmission was independent of when treatment was started (as long as it started within the first 5 days of treatment). In reality, it is possible that antiviral treatments might have different effects in reducing overall transmission depending on when during the course of an infection they are taken, e.g. reducing overall viral load and hence transmission if taken early on but having only a modest effect as time goes by. Because studies have shown contradictory results regarding the effect of vaccines in reducing infectiousness [42–45], we conservatively assumed that the vaccine had no effect in reducing infectiousness. If vaccination does reduce infectiousness then the synergy between antiviral and vaccine might be less than reported here. We assumed no further vaccination during the period of our analysis, which corresponds to the current situation in most middle- and high-vaccinated countries, where the adult population who are willing to be vaccinated and boosted has mostly been immunized. Nevertheless, vaccines for children younger than 5 years old are currently in clinical trials, and are expected to be available during summer 2022. Vaccinating this age group will increase the overall proportion of the population vaccinated in high-income countries, hence reducing further on-going transmission. In addition, WHO recently released an ambitious plan to vaccinate 70% of the global population by mid 2022 [46]. Our model was calibrated to a population in the United States and we assumed no difference in the transmission parameters for the other populations but it is possible that these parameters are location-dependent. Because we modeled four different countries with different non-pharmaceutical interventions and different cultures, we did not model any additional interventions, such as masking, or behavioral changes among diagnosed people. For simplicity, we did not include waning immunity, but explored scenarios where a higher or lower proportions of the population are currently immune. While it is extremely hard to predict when and how different populations will become vaccinated and the percentage of pre-existing immunity in each population, we believe that the scenarios that we have considered here, with different combinations of vaccination coverage and proportions of the population with pre-existing infection-induced immunity, are sufficient to capture the potential population-level effect of the potential use of antiviral treatment on transmission and COVID-19-related severe outcomes.

## Conclusions

Taken together, our results suggest that antiviral treatment can be an extremely useful tool to reduce COVID-19 related deaths and to alleviate the COVID-19 burden on overwhelmed and exhausted healthcare systems. In particular, antiviral treatments, especially oral antiviral drugs that can be easily distributed, can have a huge impact in countries which have had less access to vaccines and boosters. Further, our model shows that the population level impact of antiviral treatments are enhanced by their synergistic use in combination with vaccination, particularly in the presence of less transmissible variants. Our results suggest that in the face of highly transmissible variants, unless antiviral treatments are shown to protect against or diminish long COVID conditions, antiviral treatment is best used by targeting it to groups being at high risk for disease progression. As more data emerges, mathematical modeling can be extremely useful to determine the optimal use of antiviral treatments.

## Methods

### Main model

We adapted a previously developed agent-based transmission model, COVASIM [24]. Namely, we extended the model to include the use of antiviral treatments in the population. We briefly describe here the main features of this model (as were used in the present work) and we describe in full detail the adaptations we made. We refer to the original article by Kerr et al. [24] for the full details of the model implementation. This is an agent-based model that simulates SARS-CoV-2 transmission and interventions that was originally calibrated to data for Seattle, WA, US. Each individual in the population is modeled as an agent in a network, with 500,000 agents. The model has four possible contact layers: home, school, work or community. We selected four countries, Kenya, Mexico, US and Belgium based on two reasons: first, the availability of age-stratified data for the vaccination proportions and also because these countries can be used as representatives of (i) a very young country with a very small vaccination coverage (Kenya), (ii) a young country with a medium vaccination coverage (Mexico) and (iii) countries with an older population structure where a high proportion of the population has been vaccinated (US and Belgium). For each location, the model uses country-specific demographics and household sizes, obtained from the United Nations World Statistics Division population prospect website [47, 48]. For the age-specific targeted interventions, we then calculated the proportion of people aged 60 to 65 among those aged 60 to 70 years old [49]. The model simulates infections, interventions antiviral treatment and vaccination. We did 100 runs for each scenario. We report the median of these runs and the 10th and 90th percentiles for the lower and upper bounds.

### Disease dynamics

Individuals in the network can be susceptible, exposed (infected but not infectious), asymptomatic, presymptomatic or symptomatic. Symptomatic individuals have one of three fates: they develop a mild, severe, or critical disease. All mild infected individuals recover, and infected individuals reaching a critical state can recover or die. The latent period is sampled from a log-normal distributions with a mean of 4.6 days [24, 50]. The length of time between becoming infectious and developing symptoms is sampled from a log-normal distribution with mean 1.1 days [24, 51]. The times to develop severe symptoms, to progress to become critically ill and to death are sampled from lognormal distributions with means 6.6 [24, 51, 52], 1.5 [24, 52, 53] and 10.7 days [24, 54] respectively. Asymptomatic and mild cases recover on average on 8 days (sampled from log-normal distributions) [24, 55], while severe and critically ill cases recover on average on 18.1 days [24, 54]. Infectiousness was assumed to be linearly correlated to viral load [56]. As with the original model [24], we modeled viral load having two modes: first, a high mode where viral load is highest, around or before symptom onset, and a low mode having a longer duration but a lower viral load (50% lower than during the high mode). We assumed that children are less likely to develop symptoms than adults but equally likely to become infected [57, 58], Additional file 1: Table S3. Further, asymptomatic individuals are assumed to be 30% less infectious than symptomatic individuals [59].

### Viral transmission

We assumed that antiviral treatment would be deployed under epidemic waves with characteristics similar to the Delta or the Omicron waves. For the Delta wave, we assumed an increased transmissibility of 97% with respect to the ancestral variant [5]. For the Omicron wave, we assumed an increased transmissibility of 66% with respect to the Delta variant [60]. These assumptions resulted in basic reproduction number $R_0$ for a Delta-like variant of approximately 4.95–5.68 and of 7.75–8.63 for an Omicron-like variant. We assumed that 0.5% of the population is infected at the beginning of the simulation and that 40% of the population (selected at random) in each country was previously infected and is immune for the duration of the study (20 and 60% were also explored in Sensitivity analysis). Additional file 1: Table S1 summarizes all the parameters used in the model.

### Antiviral treatment

As described above, we assumed that antiviral treatment had two main effects. First, antiviral treatment reduced the probability of symptomatic infected individuals becoming hospitalized. Second, we reduced viral load in treated individuals by 25, 50, 75 or 100%. Because in our model, viral load linearly correlates with infectiousness, this assumption resulted in equal reductions in the overall transmissibility of treated infected individuals. Because different countries might have different testing rates, we did not explicitly model any testing. Instead, each day of the simulation, the model identified eligible individuals (symptomatic adults over 18, 30, 50 or 65 years of age) whose symptoms onset was within the time frame studied (5 days, 2 days or within 3–5 days of symptoms onset). It then randomly selected a percentage of these individuals (excluding those who are already in treatment) for antiviral treatment initiation. Once an infected symptomatic individual initiated treatment, we assumed that antiviral effects would last for the subsequent days of his or her infection.

### Vaccination

We considered a leaky vaccine (that is, a vaccine that confers partial protection to all vaccinated individuals) [61] having three effects on vaccinated individuals: to reduce the probability of acquiring a SARS-CoV-2 infection (denoted by$\text {VE}_{\varvec{\text {SUS}}}$), to reduce the probability of developing COVID-19 symptoms after infection (denoted by $\text {VE}_{\varvec{\text {SYMP}}}$), and to reduce the probability of hospitalization conditioned on symptomatic infection (denoted by $\text {VE}_{\varvec{\text {H}}}$). Then it follows that

$$\begin{aligned} \text {VE}_{\text {DIS}}&= 1 - (1- \text {VE}_{\varvec{\text {SUS}}})(1- \text {VE}_{\varvec{\text {SYMP}}})\\ \text {VE}_{\text {HOSP}}&= 1 - (1- \text {VE}_{\text {DIS}})(1- \text {VE}_{\varvec{\text {H}}}) \end{aligned}$$

where $\text {VE}_{\text {DIS}}$ and $\text {VE}_{\text {HOSP}}$ are the unconditional vaccine effectiveness on symptomatic infection and hospitalization respectively. Importantly, under this model vaccination does not influence onward transmission of infection except through a reduction in SARS-CoV-2 infection. Vaccine effectiveness estimates for each modeled wave can be found in Additional file 1: Table S2. For Mexico, the US and Belgium, we used country- and age-specific vaccination rates (vaccination rates as of October 12th 2021 and January 3rd, 2022 for the Delta and Omicron waves respectively), Additional file 1: Tables S5–S7 [62–65]. For the US and Belgium, vaccinated individuals were further split between those having high (boosted individuals) or low protection for the Omicron wave. Since Kenya had fully vaccinated only a very small proportion of its population –1.5% and 7.7% of its population for each of these dates—–and targeted front-line health workers, teachers, police and military, we distributed its vaccine randomly among all adults [3, 65, 66].

## Supplementary Information

**Additional file 1: Table S1.** Parameters used in the model. **Table S2.** Vaccine effectiveness values used in the model during the Delta and the Omicron waves. For the Delta wave, vaccine effectiveness assumes full coverage. For the Omicron wave, we assumed that boosted vaccinated individuals will get the same protection as that given by full coverage during the Delta wave. **Table S3.** Age-specific parameters for disease progression.** Table S4.** Demographic distribution of the adult population. **Table S5.** Vaccine distribution in Belgium (values taken from [20, 21]). As of January 3rd, certain groups (e.g. children who just got vaccinated) in Belgium were were considered “highly protected” and were given the vaccine effectiveness of the boosted vaccinated individuals in the model. **Table S6.** Vaccine distribution in US (values taken from [21, 22]). As of January 3rd, children under 18 years old were considered “highly protected” and were given the vaccine effectiveness of the boosted vaccinated individuals in the model. **Table S7.** Vaccine distribution in Mexico (values taken from [21, 23]). As of January 3rd, we found no data on boosted individuals in Mexico, so we did not considered boosted individuals in Mexico [21]. **Figure S1.** Cumulative deaths over next 6 months in A) Kenya, B) Mexico, C) United States and D) Belgium. Here, we assumed an epidemic wave with parameters similar to those of the Omicron epidemic wave (transmissibility, vaccine effectiveness, and vaccination coverage). For each country, the colors represent four possible values of AVT (25, 50, 75 or 100% reduction in viral transmission in treated  symptomatic individuals) and a daily treatment initiation rate (DTIR) of 2-100% of adult symptomatic individuals within the first 5 days of symptoms. Gray bars represent baseline cumulative deaths in absence of antiviral treatment. **Figure S2.** Cumulative infections over next 6 months for A) Kenya, B) Mexico, C) United States and D) Belgium. Here, we assumed an epidemic wave with parameters similar to those of the Omicron epidemic wave (transmissibility, vaccine effectiveness, and vaccination coverage). For each country, the colors represent four possible values of AVT (25, 50, 75 or 100% reduction in viral transmission in treated symptomatic individuals) and a daily treatment initiation rate (DTIR) of 2-100% of adult symptomatic individuals within the first 5 days of symptoms. Gray bars represent baseline cumulative infections in absence of antiviral treatment. **Figure S3.** Percentage of deaths averted (compared to a baseline of no antiviral treatment) for A) Kenya, B) Mexico, C) United States and D) Belgium. Here, we assumed an epidemic wave with parameters similar to those of the Delta epidemic wave (transmissibility, vaccine effectiveness, and vaccination coverage). For each country, the colors represent four possible values of AVT (25, 50, 75 or 100% reduction in viral transmission in treated symptomatic individuals) and a daily treatment initiation rate (DTIR) of 2-100% of adult symptomatic individuals within the first 5 days of symptoms. **Figure S4.** Percentage of infections averted (compared to a baseline of no antiviral treatment) for A) Kenya, B) Mexico, C) United States and D) Belgium. Here, we assumed an epidemic wave with parameters similar to those of the Delta epidemic wave (transmissibility, vaccine effectiveness, and vaccination coverage). For each country, the colors represent four possible values of AVT (25, 50, 75 or 100% reduction in viral transmission in treated symptomatic individuals) and a daily treatment initiation rate (DTIR) of 2-100% of adult symptomatic individuals within the first 5 days of symptoms. **Figure S5.** Percentage of deaths averted for A) Kenya, B) Mexico, C) United States and D) Belgium assuming 20 (left), 40 (middle) or 60% (right) of the population has been previously infected and is currently immune. For each country, the colors represent four possible values of AVT (25, 50, 75 or 100% reduction in viral transmission in treated symptomatic individuals) and a daily treatment initiation rate (DTIR) of 2-100% of adult symptomatic individuals within the first 5 days of symptoms. **Figure S6.** Daily new infections assuming no antiviral treatment (Baseline) or assuming a daily treatment initiation rate of of 10-100% of eligible symptomatic individuals in A) Kenya, B) Mexico, C) United States and D) Belgium assuming 20% of the population has been previously infected and is now recovered. For each location, each column represents a different value of AVT (25, 50, 75 or 100% reduction in viral transmission in treated symptomatic individuals). **Figure S7.** Daily new infections assuming no antiviral treatment (Baseline) or assuming a daily treatment initiation rate of of 10-100% of eligible symptomatic individuals in A) Kenya, B) Mexico, C) United States and D) Belgium assuming 60% of the population has been previously infected and is now recovered. For each location, each column represents a different value of AVT 25, 50, 75 or 100% reduction in viral transmission in treated symptomatic individuals). **Figure S8.** Percentage of deaths averted (compared to a baseline of no antiviral treatment) for A) Kenya, B) Mexico, C) United States and D) Belgium assuming antiviral treatment would reduce hospitalizations by 30%. Here, we assumed an epidemic wave with parameters similar to those of the Omicron epidemic wave (transmissibility, vaccine effectiveness, and vaccination coverage). For each country, the colors represent four possible values of AVT (25, 50, 75 or 100% reduction in viral transmission in treated symptomatic individuals) and a daily treatment initiation rate (DTIR) of 2-100% of adult symptomatic individuals within the first 5 days of symptoms. **Figure S9.** Percentage of deaths averted (compared to a baseline of no antiviral treatment) for A) Kenya, B) Mexico, C) United States and D) Belgium assuming asymptomatic infections are 50% less infectious. Here, we assumed an epidemic wave with parameters similar to those of the Omicron epidemic wave (transmissibility, vaccine effectiveness, and vaccination coverage). For each country, the colors represent four possible values of AVT (25, 50, 75 or 100% reduction in viral transmission in treated symptomatic individuals) and a daily treatment initiation rate (DTIR) of 2-100% of adult symptomatic individuals within the first 5 days of symptoms. **Figure S10.** Percentage of infections averted (compared to a baseline of no antiviral treatment) for A) Kenya, B) Mexico, C) United States and D) Belgium assuming asymptomatic infections are 50% less infectious. Here, we assumed an epidemic wave with parameters similar to those of the Omicron epidemic wave (transmissibility, vaccine effectiveness, and vaccination coverage). For each country, the colors represent four possible values of AVT (25, 50, 75 or 100% reduction in viral transmission in treated symptomatic individuals) and a daily treatment initiation rate (DTIR) of 2-100% of adult symptomatic individuals within the first 5 days of symptoms.

## Data Availability

All data Code available at: https://github.com/lulelita/COVID19Antivirals.

## Abbreviation

DTIR: Daily treatment initiation rate
