# Supplementary material for: Could widespread use of antiviral treatment curb the COVID-19 pandemic? A modeling study
Source: BMC Infect Dis. 2022 Aug 9;22:683. doi: 10.1186/s12879-022-07639-1 (PMC9361252; doi:10.1186/s12879-022-07639-1)
Supplement: Supplementary file 1 — Additional file 1: Table S1. Parameters used in the model. Table S2. Vaccine effectiveness values used in the model during the Delta and the Omicron waves. For the Delta wave, vaccine effectiveness assumes full coverage. For the Omicron wave, we assumed that boosted vaccinated individuals will get the same protection as that given by full coverage during the Delta wave. Table S3. Age-specific parameters for disease progression. Table S4. Demographic distribution of the adult population. Table S5. Vaccine distribution in Belgium (values taken from [20, 21]). As of January 3rd, certain groups (e.g. children who just got vaccinated) in Belgium were were considered “highly protected” and were given the vaccine effectiveness of the boosted vaccinated individuals in the model. Table S6. Vaccine distribution in US (values taken from [21, 22]). As of January 3rd, children under 18 years old were considered “highly protected” and were given the vaccine effectiveness of the boosted vaccinated individuals in the model. Table S7. Vaccine distribution in Mexico (values taken from [21, 23]). As of January 3rd, we found no data on boosted individuals in Mexico, so we did not considered boosted individuals in Mexico [21]. Figure S1. Cumulative deaths over next 6 months in A) Kenya, B) Mexico, C) United States and D) Belgium. Here, we assumed an epidemic wave with parameters similar to those of the Omicron epidemic wave (transmissibility, vaccine effectiveness, and vaccination coverage). For each country, the colors represent four possible values of AVT (25, 50, 75 or 100% reduction in viral transmission in treated symptomatic individuals) and a daily treatment initiation rate (DTIR) of 2-100% of adult symptomatic individuals within the first 5 days of symptoms. Gray bars represent baseline cumulative deaths in absence of antiviral treatment. Figure S2. Cumulative infections over next 6 months for A) Kenya, B) Mexico, C) United States and D) Belgium. Here, we assumed an epidemic wav [file 12879_2022_7639_MOESM1_ESM.pdf]

Additional Information: Could widespread use of antiviral treatment curb the COVID-19 pandemic? A modeling study.

Laura Matrajt,[1*] Elizabeth R. Brown[1,2], Myron S. Cohen,[3], Dobromir Dimitrov[1,4], Holly Janes,[1]
[1]Vaccine and Infectious Disease Division, Fred Hutchinson Cancer Research Center, Seattle, WA, USA
[2]Department of Biostatistics, University of Washington, Seattle, WA, USA
[3] Institute of Global Health and Infectious Diseases, University of North Carolina at Chapel Hill, Chapel Hill, NC, USA
[4]Department of Applied Mathematics, University of Washington, Seattle, WA, USA
*To whom correspondence should be addressed; E-mail: laurama@fredhutch.org

Additional Tables

Table S1: Parameters used in the model.

Parameter	Distribution (mean, SD)	Reference
Latent period	lognormal(4.5, 1.5)	[1, 2]
Infectious period for asymptomatic and mild cases	lognormal(8, 2)	[1, 3]
Duration of presymptomatic period	lognormal(1.1, 0.9)	[1, 4]
Length of time from symptom onset to hospitalization	lognormal(6.6, 4.9)	[1, 4, 5]
Length of time from hospitalization to critically ill	lognormal(1.5, 1)	[1, 5, 6]
Length of time from critically ill to death	lognormal(10.7, 4.8)	[1, 7]
Time from onset of symptoms to recovery for severe and critically ill cases	lognormal(18.1, 6.3)	[1, 7]
Age-stratified mortality rates	varied by age	[1, 8, 9]
Age-stratified probability of developing symptoms	Table S3	[1, 7, 10]
Fraction of symptomatic infections <15 year old	0.25	[11]
Fraction of symptomatic infections ≥15 year old	0.6	[12–14]
Antiviral effect on viral transmission, AVT	25, 50, 75, 100	assumed
Antiviral effect on hospitalization, AVH,	50 or 80	[15, 16]

Table S2: Vaccine effectiveness values used in the model during the Delta and the Omicron waves. For the Delta wave, vaccine effectiveness assumes full coverage. For the Omicron wave, we assumed that boosted vaccinated individuals will get the same protection as that given by full coverage during the Delta wave.

Parameter	Delta	Omicron (full coverage)	Omicron (boosted)	References
Vaccine efficacy against infection, VE_{SUS}	59.6	10	59.6	[17–19]
Vaccine efficacy against symptomatic infection, VE_{DIS}	67.5	0.15	67.5	[17, 19]
Vaccine efficacy against Hospitalization, VE_{HOSP}	87.65	0.52	87.65	[17, 19]

Table S3: Age-specific parameters for disease progression.

Parameter	0-9	10-19	20-29	30-39	40-49	50-59	60-69	70-79	80-89	90+
Probability of severe disease	0.0005	0.00165	0.0072	0.0208	0.0343	0.0765	0.1328	0.20655	0.2457	0.2457
Probability of critical disease	0.00003	0.00008	0.00036	0.00104	0.00216	0.00933	0.03639	0.08923	0.1742	0.1742
Probability of death	0.00002	0.00002	0.0001	0.00032	0.00098	0.00265	0.00766	0.02439	0.08292	0.1619

Table S4: Demographic distribution of the adult population

Location	Kenya	Mexico	US	Belgium
Percentage of adults ≥ 18	100	100	100	100
Percentage of adults ≥ 30	44	59	70	74
Percentage of adults ≥ 50	13	26	40	44
Percentage of adults ≥ 65	3	9	19	22

Table S5: Vaccine distribution in Belgium (values taken from [20, 21]). As of January 3rd, certain groups (e.g. children who just got vaccinated) in Belgium were were considered "highly protected" and were given the vaccine effectiveness of the boosted vaccinated individuals in the model.

Age group	Delta wave (full dosage)	Omicron wave (full dosage)	Omicron (highly protected)
12-15	0.6975	0.0194	0.74
16-17	0.7886	0.0351	0.81
18-24	0.7924	0.0805	0.76
25-34	0.7884	0.0809	0.75
35-44	0.8369	0.1257	0.74
45-54	0.887	0.2148	0.69
55-64	0.9154	0.1769	0.75
65-74	0.9317	0.09	0.85
75-85	0.9378	0.0753	0.87
$\geq$85	0.9139	0.11	0.81

Table S6: Vaccine distribution in US (values taken from [21, 22]). As of January 3rd, children under 18 years old were considered "highly protected" and were given the vaccine effectiveness of the boosted vaccinated individuals in the model.

Age group	Delta wave (full dosage)	Omicron wave (full dosage)	Omicron (highly protected)
5-11	-	-	0.156
12-15	0.446	-	0.536
16-17	0.524	-	0.536
18-24	0.528	0.389	0.20
25-39	0.57	0.417	0.215
40-49	0.658	0.471	0.243
50-64	0.737	0.431	0.353
65-74	0.86	0.36	0.539
$\geq$75	0.809	0.338	0.506

Table S7: Vaccine distribution in Mexico (values taken from [21, 23]). As of January 3rd, we found no data on boosted individuals in Mexico, so we did not considered boosted individuals in Mexico [21].

Age group	Delta wave (full dosage)	Omicron wave (full dosage)
18-39	0.493	0.83
40-49	0.735	0.735
50-59	0.785	0.785
60	0.824	0.824

Additional Figures

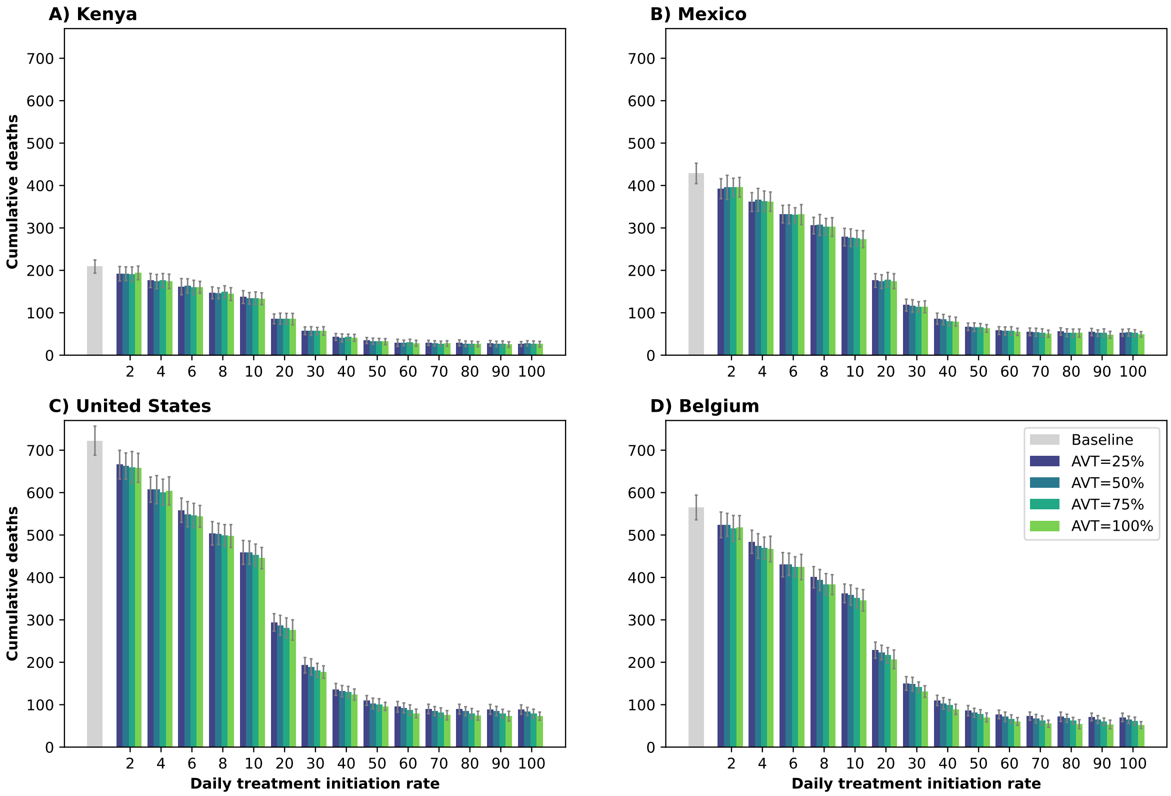

Figure S1: Cumulative deaths over next 6 months in A) Kenya, B) Mexico, C) United States and D) Belgium. Here, we assumed an epidemic wave with parameters similar to those of the Omicron epidemic wave (transmissibility, vaccine effectiveness, and vaccination coverage). For each country, the colors represent four possible values of AVT (25, 50, 75 or 100% reduction in viral transmission in treated symptomatic individuals) and a daily treatment initiation rate (DTIR) of 2-100% of adult symptomatic individuals within the first 5 days of symptoms. Gray bars represent baseline cumulative deaths in absence of antiviral treatment.

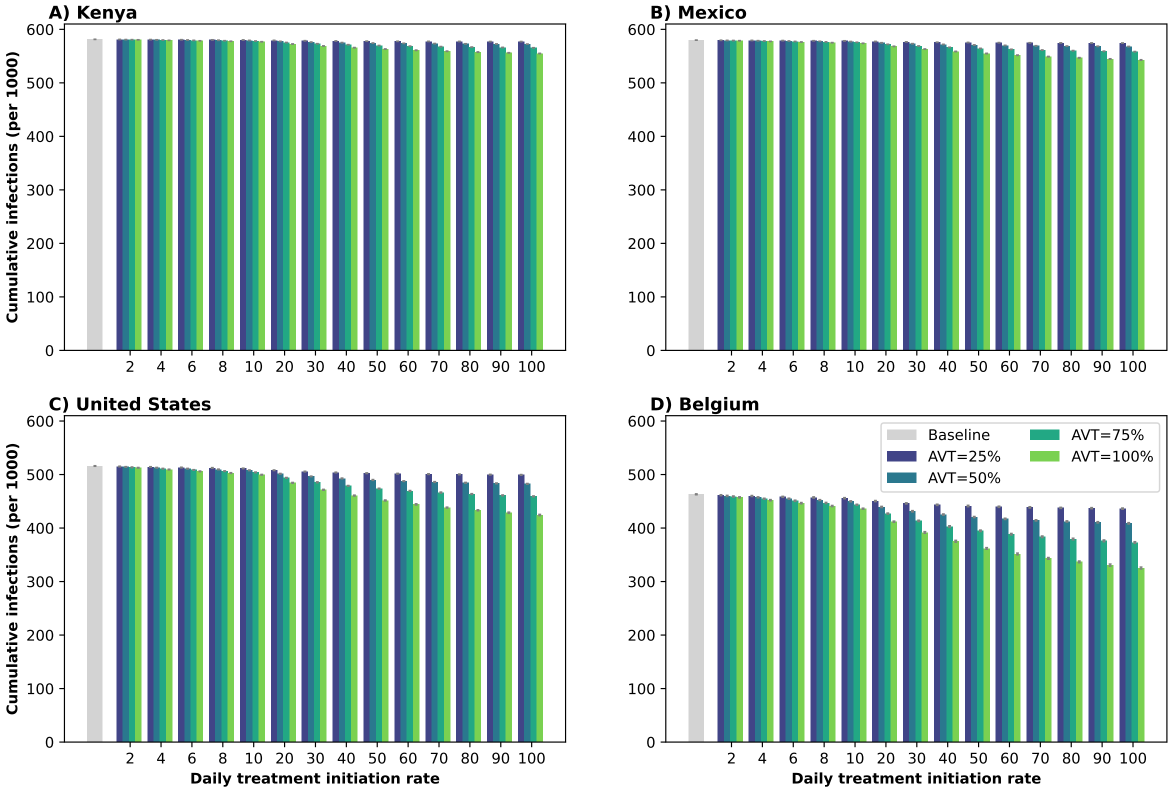

Figure S2: Cumulative infections over next 6 months for A) Kenya, B) Mexico, C) United States and D) Belgium. Here, we assumed an epidemic wave with parameters similar to those of the Omicron epidemic wave (transmissibility, vaccine effectiveness, and vaccination coverage). For each country, the colors represent four possible values of AVT (25, 50, 75 or 100% reduction in viral transmission in treated symptomatic individuals) and a daily treatment initiation rate (DTIR) of 2-100% of adult symptomatic individuals within the first 5 days of symptoms. Gray bars represent baseline cumulative infections in absence of antiviral treatment.

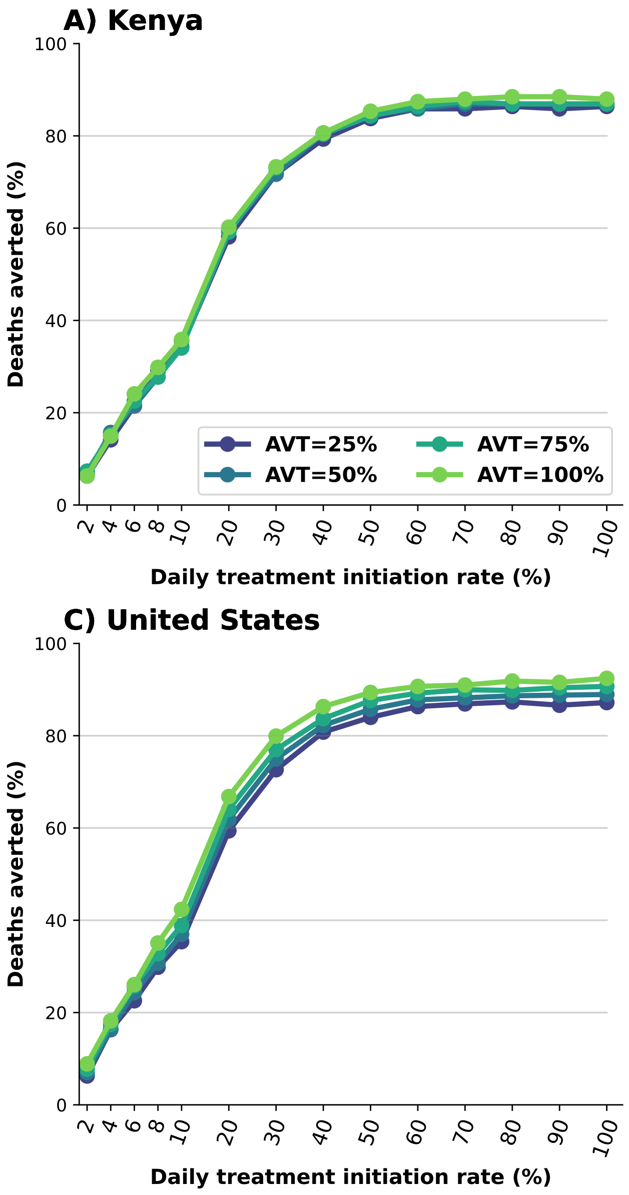

Figure S3: Percentage of deaths averted (compared to a baseline of no antiviral treatment) for A) Kenya, B) Mexico, C) United States and D) Belgium. Here, we assumed an epidemic wave with parameters similar to those of the Delta epidemic wave (transmissibility, vaccine effectiveness, and vaccination coverage). For each country, the colors represent four possible values of AVT (25, 50, 75 or 100% reduction in viral transmission in treated symptomatic individuals) and a daily treatment initiation rate (DTIR) of 2-100% of adult symptomatic individuals within the first 5 days of symptoms.

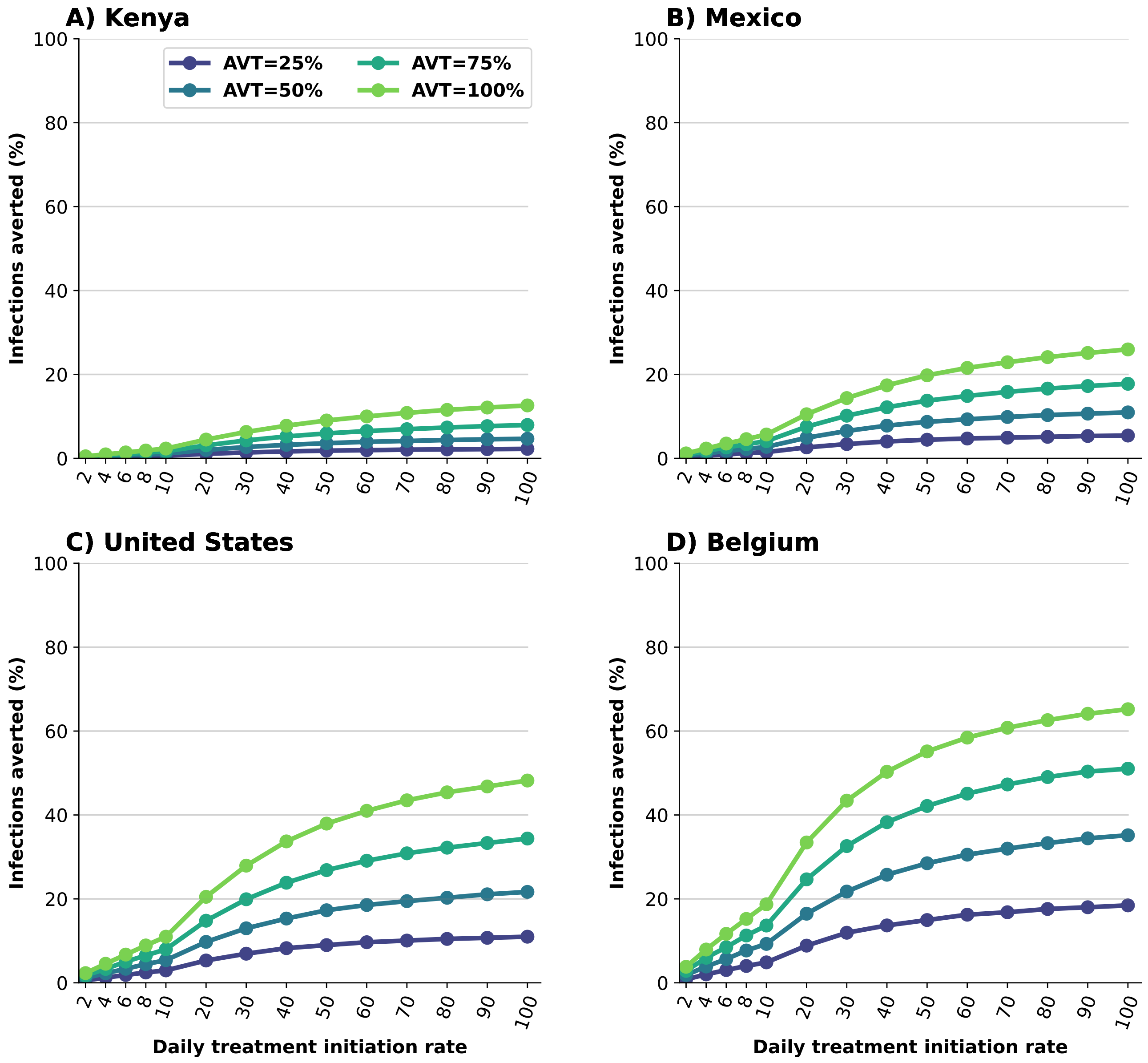

Figure S4: Percentage of infections averted (compared to a baseline of no antiviral treatment) for A) Kenya, B) Mexico, C) United States and D) Belgium. Here, we assumed an epidemic wave with parameters similar to those of the Delta epidemic wave (transmissibility, vaccine effectiveness, and vaccination coverage). For each country, the colors represent four possible values of AVT (25, 50, 75 or 100% reduction in viral transmission in treated symptomatic individuals) and a daily treatment initiation rate (DTIR) of 2-100% of adult symptomatic individuals within the first 5 days of symptoms.

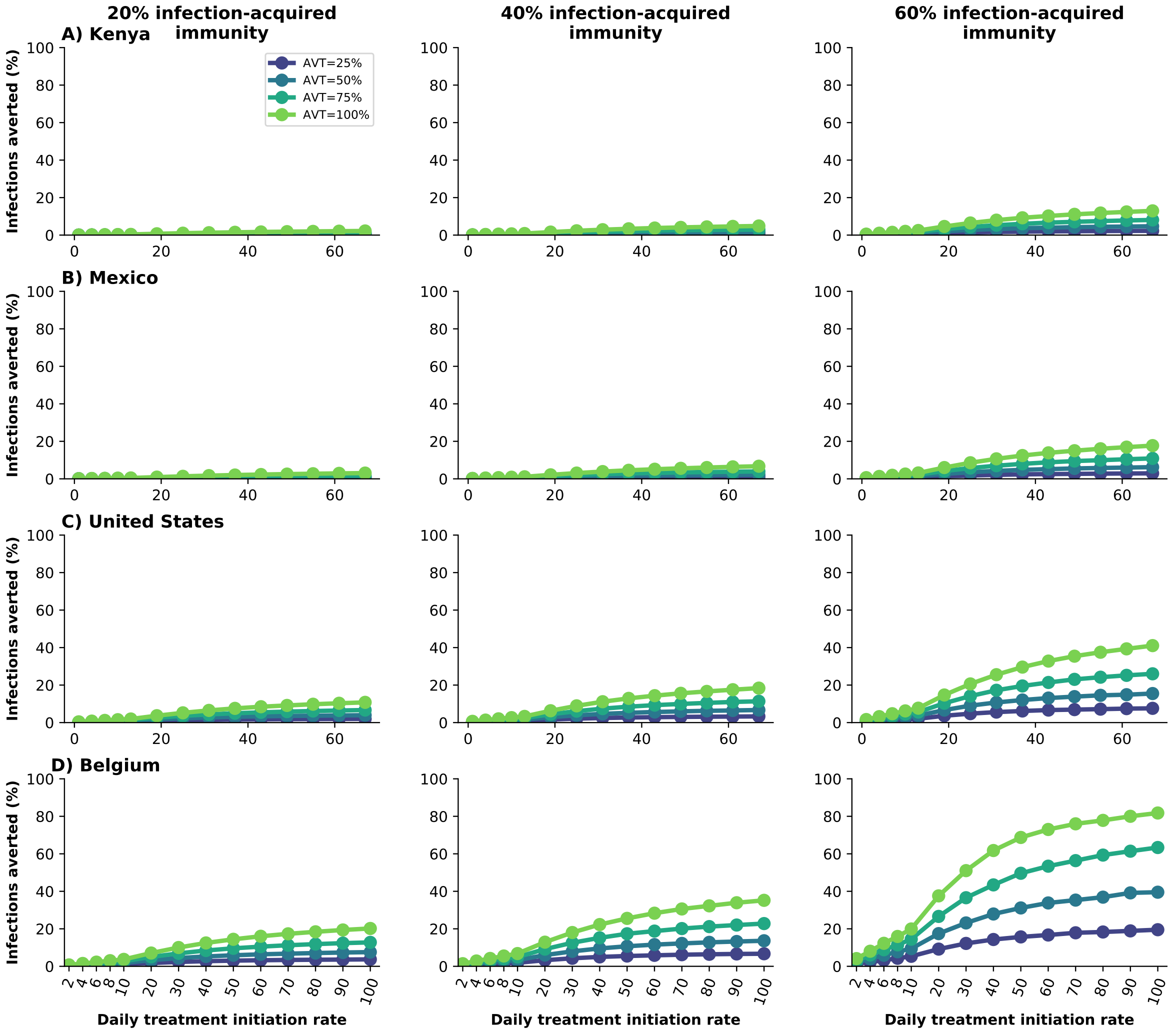

Figure S5: Percentage of deaths averted for A) Kenya, B) Mexico, C) United States and D) Belgium assuming 20 (left), 40 (middle) or 60% (right) of the population has been previously infected and is currently immune. For each country, the colors represent four possible values of AVT (25, 50, 75 or 100% reduction in viral transmission in treated symptomatic individuals) and a daily treatment initiation rate (DTIR) of 2-100% of adult symptomatic individuals within the first 5 days of symptoms.

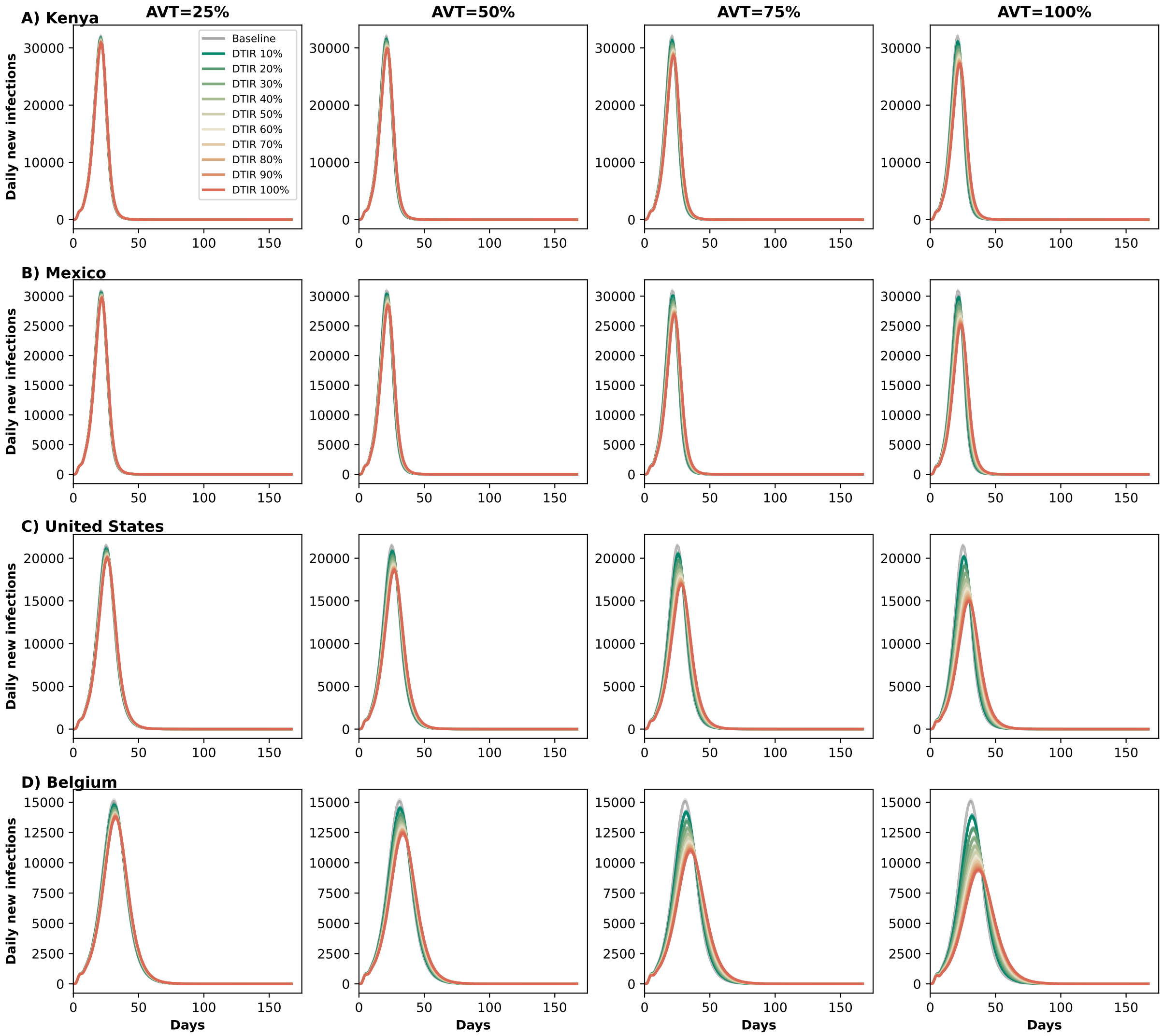

Figure S6: Daily new infections assuming no antiviral treatment (Baseline) or assuming a daily treatment initiation rate of of 10-100% of eligible symptomatic individuals in A) Kenya, B) Mexico, C) United States and D) Belgium assuming 20% of the population has been previously infected and is now recovered. For each location, each column represents a different value of AVT (25, 50, 75 or 100% reduction in viral transmission in treated symptomatic individuals).

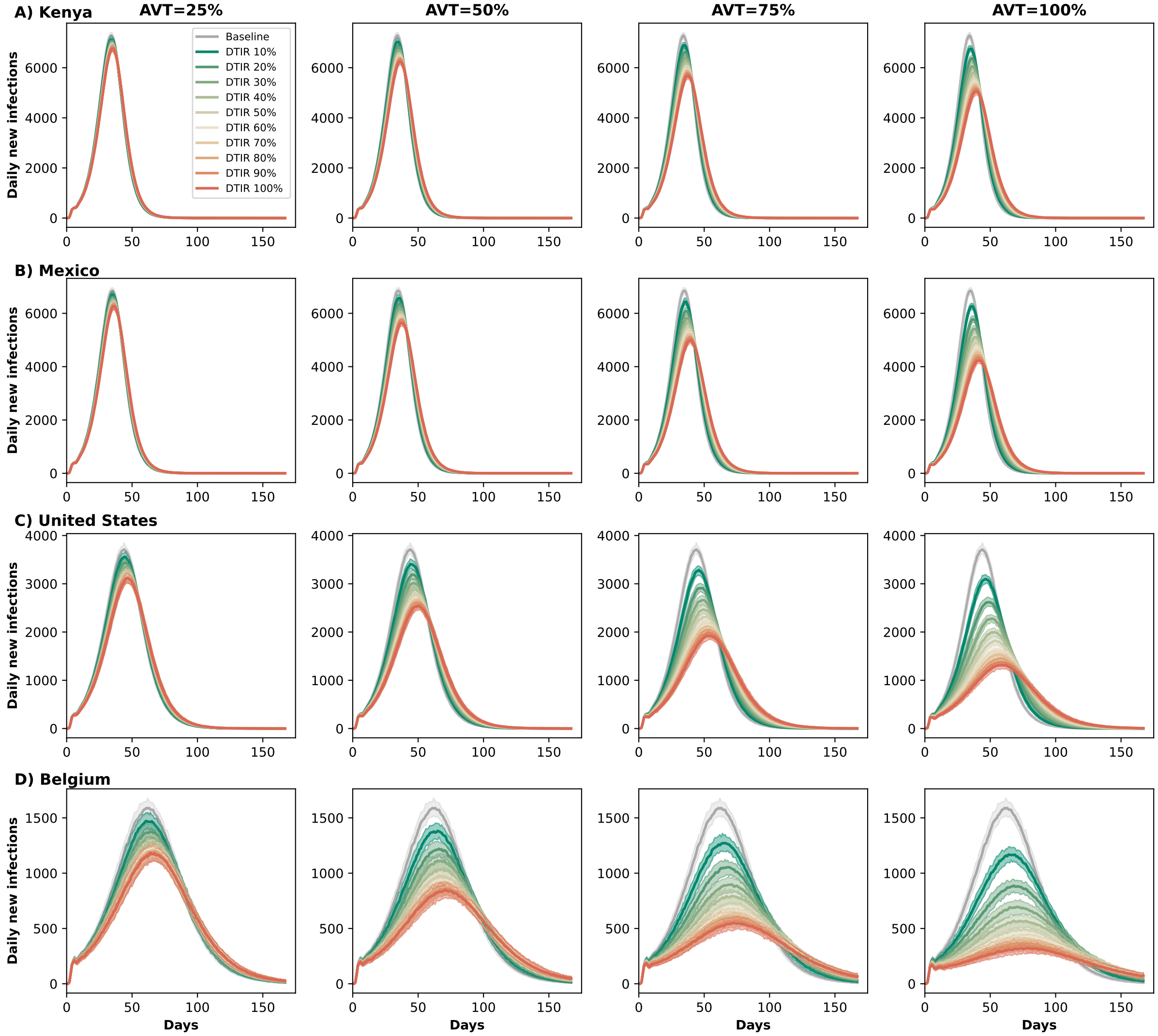

Figure S7: Daily new infections assuming no antiviral treatment (Baseline) or assuming a daily treatment initiation rate of of 10-100% of eligible symptomatic individuals in A) Kenya, B) Mexico, C) United States and D) Belgium assuming 60% of the population has been previously infected and is now recovered. For each location, each column represents a different value of AVT 25, 50, 75 or 100% reduction in viral transmission in treated symptomatic individuals).

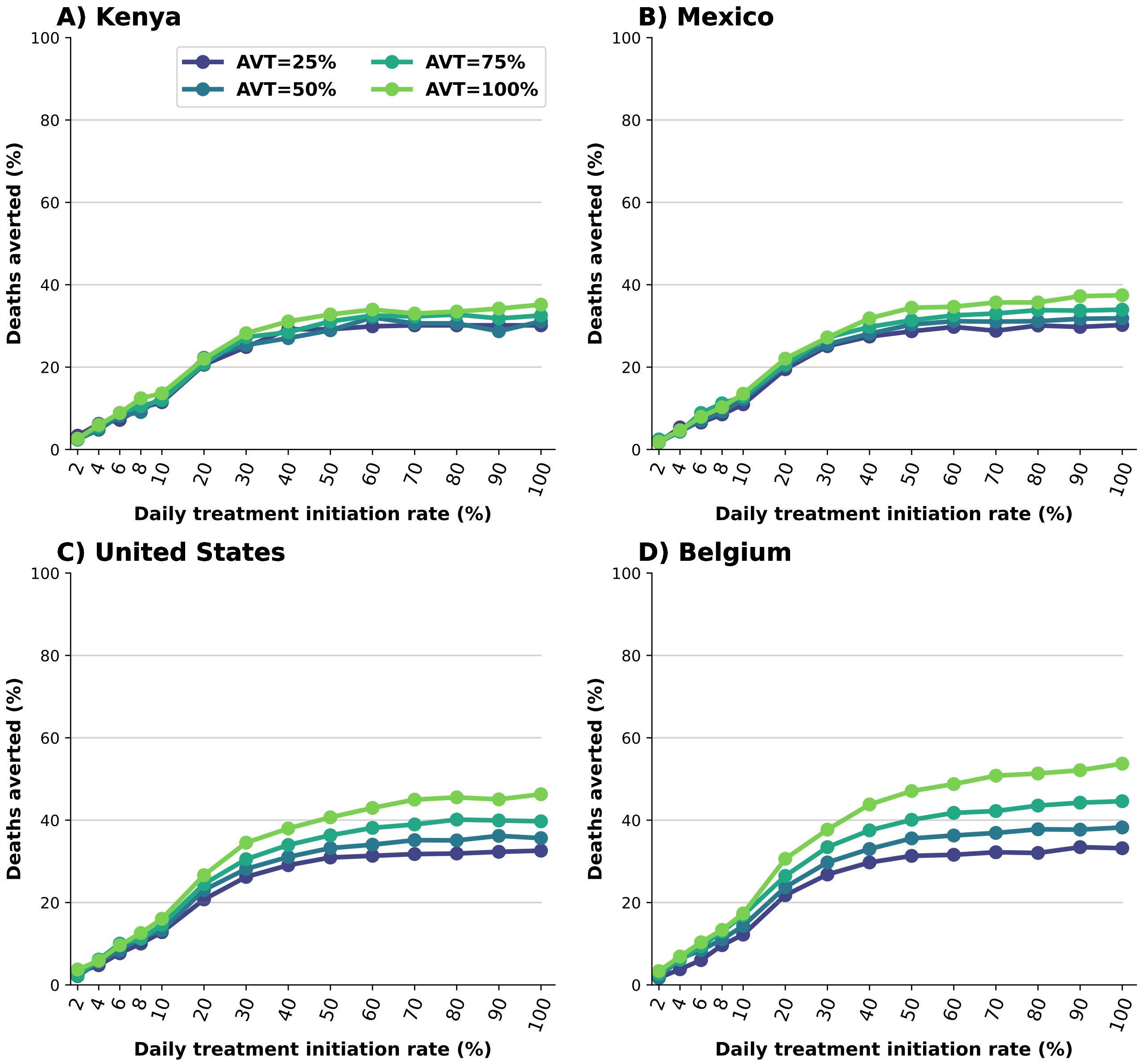

Figure S8: Percentage of deaths averted (compared to a baseline of no antiviral treatment) for A) Kenya, B) Mexico, C) United States and D) Belgium assuming antiviral treatment would reduce hospitalizations by 30%. Here, we assumed an epidemic wave with parameters similar to those of the Omicron epidemic wave (transmissibility, vaccine effectiveness, and vaccination coverage). For each country, the colors represent four possible values of AVT (25, 50, 75 or 100% reduction in viral transmission in treated symptomatic individuals) and a daily treatment initiation rate (DTIR) of 2-100% of adult symptomatic individuals within the first 5 days of symptoms.

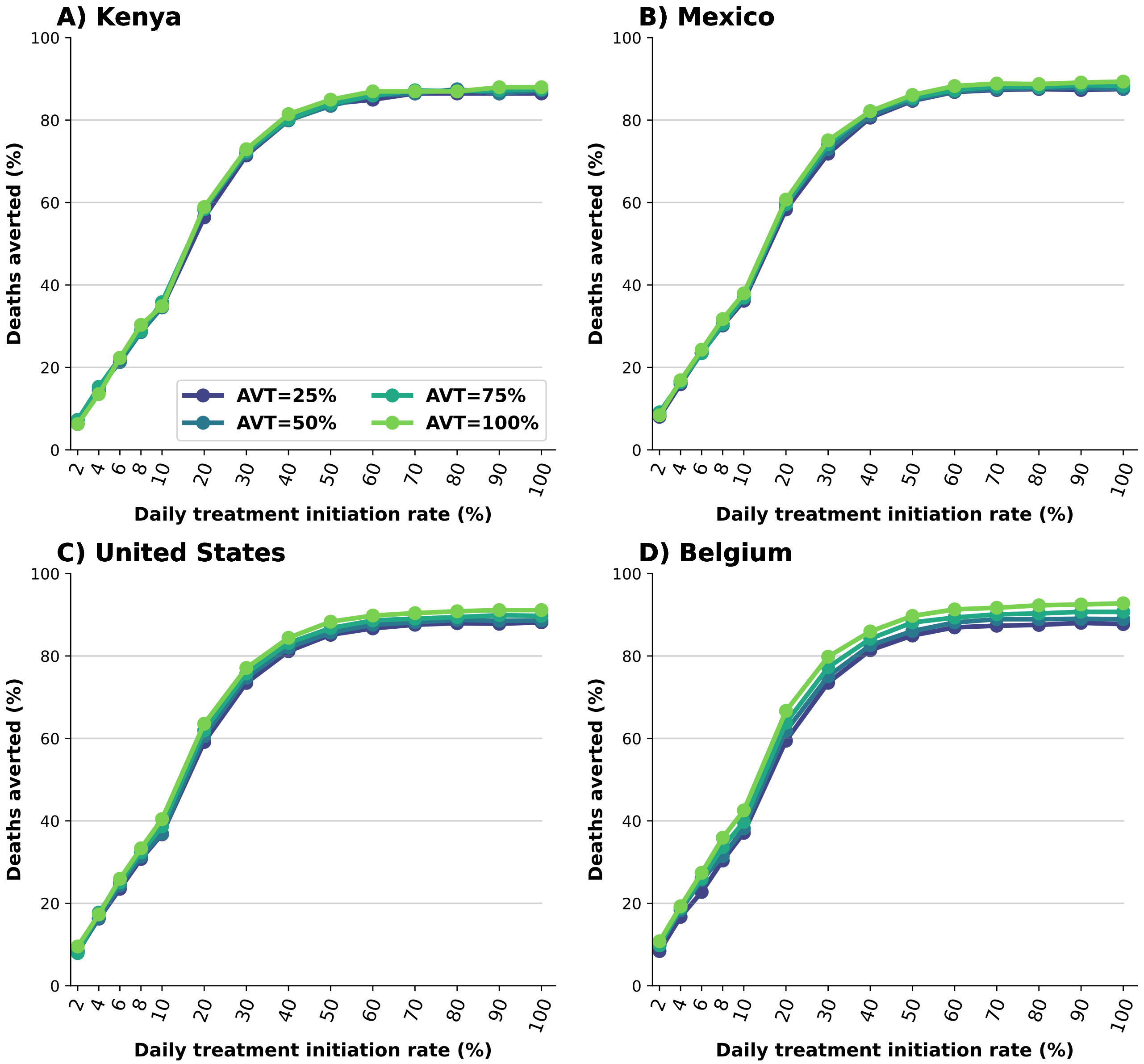

Figure S9: Percentage of deaths averted (compared to a baseline of no antiviral treatment) for A) Kenya, B) Mexico, C) United States and D) Belgium assuming asymptomatic infections are 50% less infectious. Here, we assumed an epidemic wave with parameters similar to those of the Omicron epidemic wave (transmissibility, vaccine effectiveness, and vaccination coverage). For each country, the colors represent four possible values of AVT (25, 50, 75 or 100% reduction in viral transmission in treated symptomatic individuals) and a daily treatment initiation rate (DTIR) of 2-100% of adult symptomatic individuals within the first 5 days of symptoms.

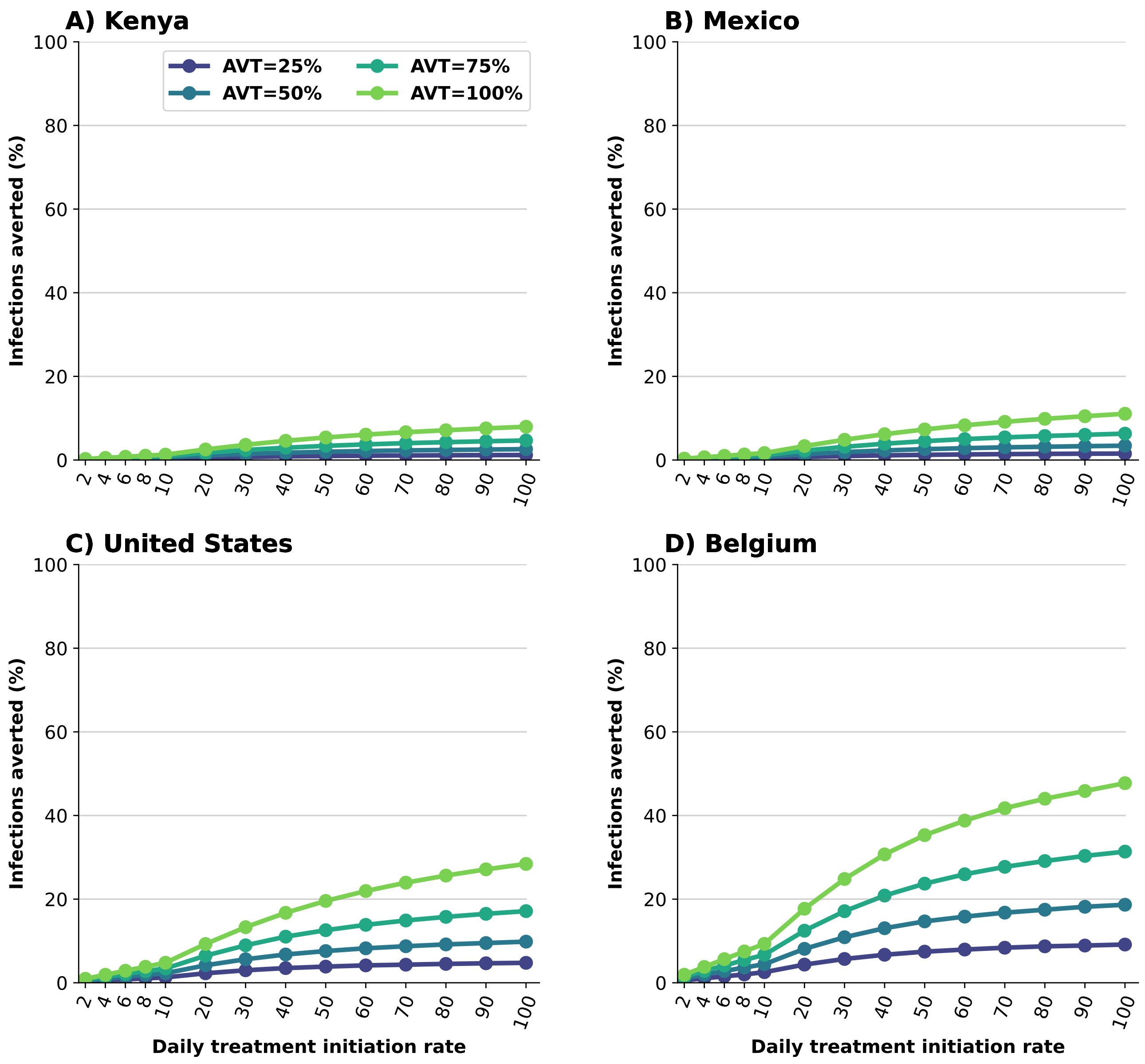

Figure S10: Percentage of infections averted (compared to a baseline of no antiviral treatment) for A) Kenya, B) Mexico, C) United States and D) Belgium assuming asymptomatic infections are 50% less infectious. Here, we assumed an epidemic wave with parameters similar to those of the Omicron epidemic wave (transmissibility, vaccine effectiveness, and vaccination coverage). For each country, the colors represent four possible values of AVT (25, 50, 75 or 100% reduction in viral transmission in treated symptomatic individuals) and a daily treatment initiation rate (DTIR) of 2-100% of adult symptomatic individuals within the first 5 days of symptoms.

References

[1] Cliff C. Kerr, Robyn M. Stuart, Dina Mistry, Romesh G. Abeysuriya, Katherine Rosenfeld, Gregory R. Hart, Rafael C. Núñez, Jamie A. Cohen, Prashanth Selvaraj, Brittany Hagedorn, Lauren George, Michał Jastrzębski, Amanda S. Izzo, Greer Fowler, Anna Palmer, Dominic Delport, Nick Scott, Sherrie L. Kelly, Caroline S. Bennette, Bradley G. Wagner, Stewart T. Chang, Assaf P. Oron, Edward A. Wenger, Jasmina Panovska-Griffiths, Michael Famulare, and Daniel J. Klein. Covasim: An agent-based model of covid-19 dynamics and interventions. *PLOS Computational Biology*, 17(7):1–32, 07 2021. doi: 10.1371/journal.pcbi.1009149. URL `https://doi.org/10.1371/journal.pcbi.1009149`.

[2] Stephen A Lauer, Kyra H Grantz, Qifang Bi, Forrest K Jones, Qulu Zheng, Hannah R Meredith, Andrew S Azman, Nicholas G Reich, and Justin Lessler. The incubation period of coronavirus disease 2019 (COVID-19) from publicly reported confirmed cases: Estimation and application. *Annals of Internal Medicine*, 2020. ISSN 1539-3704. doi: 10.7326/M20-0504. URL `http://www.ncbi.nlm.nih.gov/pubmed/32150748`.

[3] Roman Wölfel, Victor M. Corman, Wolfgang Guggemos, Michael Seilmaier, Sabine Zange, Marcel A. Müller, Daniela Niemeyer, Terry C. Jones, Patrick Vollmar, Camilla Rothe, Michael Hoelscher, Tobias Bleicker, Sebastian Brünink, Julia Schneider, Rosina Ehmann, Katrin Zwirglmaier, Christian Drosten, and Clemens Wendtner. Virological assessment of hospitalized patients with covid-2019. *Nature*, 581(7809):465–469, 2020. doi: 10.1038/s41586-020-2196-x. URL `https://doi.org/10.1038/s41586-020-2196-x`.

[4] Natalie M Linton, Tetsuro Kobayashi, Yichi Yang, Katsuma Hayashi, Andrei R Akhmetzhanov, Sung-Mok Jung, Baoyin Yuan, Ryo Kinoshita, and Hiroshi Nishiura. Incubation period and other epidemiological characteristics of 2019 novel coronavirus infections with right truncation: A statistical analysis of publicly available case data. *Journal of clinical medicine*, 9(2):538, 02 2020. doi: 10.3390/jcm9020538. URL `https://pubmed.ncbi.nlm.nih.gov/32079150`.

[5] Dawei Wang, Bo Hu, Chang Hu, Fangfang Zhu, Xing Liu, Jing Zhang, Binbin Wang, Hui Xiang, Zhenshun Cheng, Yong Xiong, Yan Zhao, Yirong Li, Xinghuan Wang, and Zhiyong Peng. Clinical Characteristics of 138 Hospitalized Patients With 2019 Novel Coronavirus–Infected Pneumonia in Wuhan, China. *JAMA*, 323(11):1061–1069, 03 2020. ISSN 0098-7484. doi: 10.1001/jama.2020.1585. URL `https://doi.org/10.1001/jama.2020.1585`.

[6] Jun Chen, Tangkai Qi, Li Liu, Yun Ling, Zhiping Qian, Tao Li, Feng Li, Qingnian Xu, Yuyi Zhang, Shuibao Xu, Zhigang Song, Yigang Zeng, Yinzhong Shen, Yuxin Shi, Tongyu Zhu, and Hongzhou Lu. Clinical progression of patients with covid-19 in shanghai, china. *Journal of Infection*, 80(5):e1–e6, 2020. ISSN 0163-4453. doi: https://doi.org/10.1016/j.jinf.2020.03.004. URL `https://www.sciencedirect.com/science/article/pii/S0163445320301195`.

[7] Robert Verity, Lucy C Okell, Ilaria Dorigatti, Peter Winskill, Charles Whittaker, Natsuko Imai, Gina Cuomo-Dannenburg, Hayley Thompson, Patrick G T Walker, Han Fu, Amy Dighe, Jamie T Griffin, Marc Baguelin, Sangeeta Bhatia, Adhiratha Boonyasiri, Anne Cori, Zulma Cucunubá, Rich FitzJohn, Katy Gaythorpe, Will Green, Arran Hamlet, Wes Hinsley, Daniel Laydon, Gemma Nedjati-Gilani, Steven Riley, Sabine van Elsland, Erik Volz, Haowei Wang, Yuanrong Wang, Xiaoyue Xi, Christl A Donnelly, Azra C Ghani, and Neil M Ferguson. Estimates of the severity of coronavirus disease 2019: a model-based analysis. *The Lancet Infectious Diseases*, 20(6):669–677, 2021/11/01 2020. doi: 10.1016/S1473-3099(20)30243-7. URL `https://doi.org/10.1016/S1473-3099(20)30243-7`.

[8] Megan O'Driscoll, Gabriel Ribeiro Dos Santos, Lin Wang, Derek A. T. Cummings, Andrew S. Azman, Juliette Paireau, Arnaud Fontanet, Simon Cauchemez, and Henrik Salje. Age-specific mortality and immunity patterns of sars-cov-2. *Nature*, 590(7844):140–145, 2021. doi: 10.1038/s41586-020-2918-0. URL `https://doi.org/10.1038/s41586-020-2918-0`.

[9] Nick F Brazeau, Robert Verity, Sara Jenks, Han Fu, Charles Whittaker, Pete Winskill, Ilaria Dorigatti, Patrick G T Walker, Steven Riley, Ricardo P Schnekenbert, Henrique Hoeltgebaum, Thomas A Mellan, Swapnil Mishra, H Juliette T Unwin, Oliver J Watson, Zulma Cucunuba, Marc Baguelin, Lilith K Whittles, Samir Bhatt, Azra C. Ghani, Ferguson Neil M., and Lucy C Okell. Report 34 - COVID-19 Infection Fatality Ratio Estimates from Seroprevalence. `https://www.imperial.ac.uk/mrc-global-infectious-disease-analysis/covid-19/report-34-ifr/` 2020.

[10] Neil M Ferguson, Daniel Laydon, Gemma Nedjati-Gilani, Natsuko Imai, Kylie Ainslie, Marc Baguelin, Sangeeta Bhatia, Adhiratha Boonyasiri, Zulma Cucunubá, Gina Cuomo-Dannenburg, Amy Dighe, Ilaria Dorigatti, Han Fu, Katy

Gaythorpe, Will Green, Arran Hamlet, Wes Hinsley, Lucy C Okell, Sabine van Elsland, Hayley Thompson, Robert Verity, Erik Volz, Haowei Wang, Yuanrong Wang, Patrick G T Walker, Caroline Walters, Peter Winskill, Charles Whittaker, Christl A Donnelly, Steven Riley, and Azra C Ghani. Impact of non-pharmaceutical interventions (NPIs) to reduce COVID-19 mortality and healthcare demand. 2020. URL https://doi.org/10.25561/77482.

[11] Russell M. Viner, Oliver T. Mytton, Chris Bonell, G. J. Melendez-Torres, Joseph Ward, Lee Hudson, Claire Waddington, James Thomas, Simon Russell, Fiona Van Der Klis, Archana Koirala, Shamez Ladhani, Jasmina Panovska-Griffiths, Nicholas G. Davies, Robert Booy, and Rosalind M. Eggo. Susceptibility to SARS-CoV-2 infection among children and adolescents compared with adults: A systematic review and meta-analysis. *JAMA Pediatrics*, 175(2):143–156, 2021. ISSN 21686211. doi: 10.1001/jamapediatrics.2020.4573.

[12] CDC. COVID-19 Pandemic Planning Scenarios. URL https://www.cdc.gov/coronavirus/2019-ncov/hcp/plannin

[13] Yong-Hoon Lee, Chae Moon Hong, Dae Hyun Kim, Taek Hoo Lee, and Jaetae Lee. Clinical course of asymptomatic and mildly symptomatic patients with coronavirus disease admitted to community treatment centers, South Korea. *Emerging Infectious Diseases*, Oct, 2020. doi: 10.3201/eid2610.201620. URL https://wwwnc.cdc.gov/eid/article/26/10/20-1620_article.

[14] Kenji Mizumoto, Katsushi Kagaya, Alexander Zarebski, and Gerardo Chowell. Estimating the asymptomatic proportion of coronavirus disease 2019 (COVID-19) cases on board the Diamond Princess cruise ship, Yokohama, Japan, 2020. *Euro Surveillance*, 25(10), 2020. URL doi.org/10.2807/1560-7917.

[15] Gilead. Veklury® (Remdesivir) Significantly Reduced Risk of Hospitalization in High-Risk Patients with COVID-19. https://www.gilead.com/news-and-press/press-room/press-releases/2021/9/veklury-remdesivir September 2021. last accessed October 19th, 2021.

[16] Merck. Merck and Ridgeback's Investigational Oral Antiviral Molnupiravir Reduced the Risk of Hospitalization or Death by Approximately 50 Percent Compared to Placebo for Patients with Mild or Moderate COVID-19 in Positive Interim Analysis of Phase 3 Study - Merc. https://www.merck.com/news/merck-and-ridgebacks-investigational-oral-antiviral-molnupirav October 2021. last accessed October 19th, 2021.

[17] Centers for Disease Control and Prevention. CDC COVID Data Tracker. https://covid.cdc.gov/covid-data-tracker/#vaccine-effectiveness, 2020. last accessed October 19th, 2021.

[18] Neil Ferguson, Azra Ghani, Anne Cori, Alexandra Hogan, Wes Hinsley, Erik Volz, and on behalf of the Imperial College COVID-19 response Team. Report 49 - Growth, population distribution and immune escape of Omicron in England | Faculty of Medicine | Imperial College London, 2021. URL https://www.imperial.ac.uk/mrc-global-infectious-disease-analysis/covid-19/report-49-Omic

[19] UK Health Security Agency. SARS-CoV-2 variants of concern and variants under investigation in England, Technical Briefing 31. https://assets.publishing.service.gov.uk/government/uploads/system/uploads/attach Dec 2021. last accessed February 24, 2022.

[20] Sciensano. Belgium COVID-19 Dashboard, Sciensano, Vaccination, 2021. URL https://datastudio.google.com/u/0/reporting/c14a5cfc-cab7-4812-848c-0369173148ab/page/hOM last accessed October 12th, 2021.

[21] OurWorldInData.org. COVID-19 vaccine doses, people with at least one dose, people fully vaccinated, and boosters per 100 people. https://ourworldindata.org/explorers/coronavirus-data-explorer, 2021. last accessed February 24, 2022.

[22] Centers for Disease Control and Prevention. COVID-19 Vaccination and Case Trends by Age Group, United States Vaccinations, 2021. URL https://data.cdc.gov/Vaccinations/COVID-19-Vaccination-and-Case-Trends-by-Age-Group-/gxj9 last accessed October 12th, 2021.

[23] Secretaria de Salud, Mexico. Vacuna COVID. http://vacunacovid.gob.mx/wordpress/, 2021. last accessed October 12th, 2021.
